# A cross sectional comparison of drug use indicators using WHO methodology in primary level hospitals participating in an Auditable Pharmaceutical Transactions and Services program versus non-APTS primary hospitals in Southern Ethiopia

**DOI:** 10.1371/journal.pone.0223523

**Published:** 2019-10-07

**Authors:** Biruk Wogayehu, Yilma Chisha, Be’emnetu Tekabe, Ayalew Adinew, Mulugeta Asefaw

**Affiliations:** 1 Department of Pharmacy, Arbaminch College of Health Sciences, Arbaminch, Ethiopia; 2 Department of Public Health, Arbaminch University, Arbaminch, Ethiopia; 3 Pharmacy Service, Federal Ministry of Health, Addis Ababa, Ethiopia; 4 Pharmacy Service, Regional Health Bureau, Hawassa, Ethiopia; College of Pharmacy & Health Sciences, UNITED STATES

## Abstract

**Introduction:**

Ethiopian pharmaceutical sector has been facing inaccessibility and unaffordability to key essential medicines due to medicines diversion from the public to private health care facilities, lack of transparency, poor inventory management, and poor dispensing workflow. In an effort to improve the pharmaceutical sector, the government of Ethiopia in 2011 introduced Auditable Pharmaceutical Transactions and Services program. This study intended to compare drug use indicators in auditable and non-auditable primary level hospitals.

**Methods:**

A cross-sectional comparative study was conducted between January 2018 and December 2018 at primary level hospitals in southern Ethiopia: one with Auditable Pharmaceutical Transactions and Services (APTS) program; another without APTS (Non-APTS).WHO drug use indicators in auditable primary hospitals (n = 10) and similar non-auditable primary hospitals (n = 10) were compared. The prescribing indicators and average cost of medicines were evaluated retrospectively using 1000 prescriptions from each group. Patient care indicators were evaluated prospectively by interviewing and observing 1000 patients from each group. Patient satisfaction was assessed by interviewing 1000 patients from each group. Health care facilities were evaluated through observation. We performed descriptive analysis, t-test, logistic regression, Mann-Whitney U test and linear regression using SPSS version 20.0.

**Results:**

The mean consultation time in auditable and non-auditable hospitals was found to be 6.5 minutes and 3.46 minutes, respectively. The average dispensing time in auditable and non-auditable hospitals was found to be 6.6 minutes and 1.02 minutes, respectively.The proportion of drugs actually dispensed was 97.59% in APTS facilities and 76.44% in the non-auditable facilities with the lowest value seen in a non-auditable facility (51.65%). The average number of drugs per prescription was 2.32 (±1.26) and 2.84 (±1.17) in auditable and non-auditable facilities, respectively. The level of patient satisfaction on the convenience of pharmacy location, information on contraindications, availability of drugs and amount of time for counseling was significantly higher in the auditable facilities than the non-auditable facilities (p<0.001).

**Conclusions:**

This study revealed that patient care indicator values, the level of patient satisfaction on the pharmacy services and health facility indicator values were significantly better in APTS than Non-APTS primary level hospitals. Most of prescribing indicators and labeling practices were not met WHO stated standard in both auditable and non-auditable facilities.This indicates that the auditable programshould include additional strategies to reverse the existing irrational prescribing and inadequate labeling practices.

## Introduction

Access to health care consisting of essential medicines is a basic human right [1, 2]. However, 67% of the world population lacks access to essential medicines [3]. Globally, studies have indicated that more than 50% of all drugs are prescribed or dispensed incorrectly and half of the patients are unable to use them properly [4]. A secondary analysis, using data from 36 middle and low-income countries, indicated that in the public health sector, availability of essential medicines ranged from 29% to 54% [5]. If essential medicines are not available in adequate amount in governmental health care facilities, patients buy medicines out-of-pocket in the retail pharmacy, which may also lead to unnecessary expenditure. Data from several low and middle-income countries revealed that prices for private patients were 9 to 25 times greater than the international reference prices for generics [5].

Like other low-income countries, Ethiopian pharmaceutical sector has faced inaccessibility and unaffordability to key essential medicines due to medicines diversion from the public to private health care facilities, lack of transparency, poor inventory management, and poor dispensing structure and work flow [6]. A study done by the World Bank in collaboration with the federal anti-corruption authority of Ethiopia also revealed that Ethiopian pharmaceutical sector was found to be the second corrupted sector in the country [7]. A national baseline assessment conducted by Ethiopian Federal Ministry of Health (FMOH) in collaboration with the Systems for Improved Access to Pharmaceuticals and Services (SIAPS) showed that the availability of key medicine was 81.5%, the average effective counseling time was only 43 seconds and labeling of medicines was poor. The national survey also indicated that only 50.5% of clients knew all four types (dose, route of administration, time of administraton and duration of treatment) of medicines’ information [8].

According to the national pharmaceutical sector assessment in 2003 indicated that availability of key medicines in the health care facilities was 70% and 85% for public health care facilities and regional medicine warehouses, respectively [6]. These percentages are lower than the World Health Organization (WHO) ideal value of 100% [9] and the 100% goal set in the Health Sector Development Plan I (HSDP- I) [10]. Unavailability of medicines in public health facilities forces patients to revert to private retail outlet pharmacies. As a result, drugs can take up more than 50% of the real cost of a visit, raising the possibility of incurring catastrophic medicine expenditures and the associated risks of falling into poverty [11, 12]. The national assessment also indicated that expire rate of medicines was 8% in public health facilities. Besides, the country figure for a mean period of stock-outs in public health care facilities was 99.2 days [6]. A study finding from eastern Ethiopia indicated that the percentage of patients satisfied with outpatient pharmacy service was 65% which was being less than other services [13].

To improve the above mentioned and other interrelated pharmaceutical sector problems Systems for Improved Access to Pharmaceuticals and Services (SIAPS) project financed by United States Agency for International Development (USAID), in collaboration with Amhara Regional Health Bureau (Northern Ethiopia), in 2011 established and piloted a set of interventions that enhance pharmaceutical services and transactions that is Auditable Pharmaceutical Transactions and Services (APTS) [14]. APTS is a package of interventions that address transparency; and accountability; access to information for decision making; quality pharmacy services; efficient use of budget, medicines and human resources. After a single pilot study conducted in a single referral hospital in northern Ethiopia (Debre Markos Referral and Teaching Hospital), by mid-2015 the program has been implemented in 19 hospitals. Selected hospitals in other regions of the country have also been implemented the program[15].

**Fig 1:**
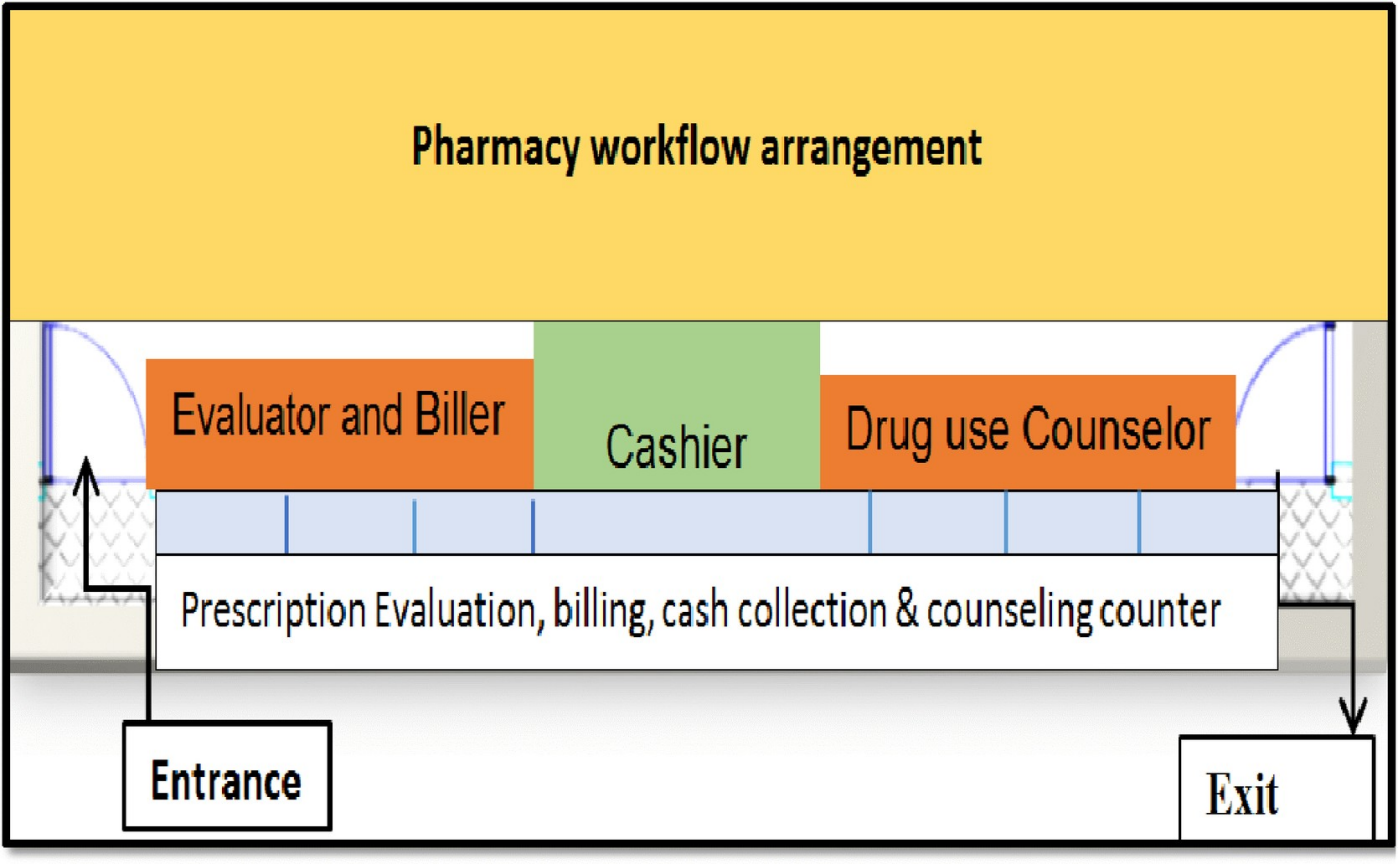
APTS pharmacy workflow arrangement (Source: APTS training guideline, 2016).

The primary purpose of the APTS is to improve patient satisfaction; accountability and transparency of pharmaceutical transactions; knowledge of prescribed medicines, production of reliable information, effective workforce deployment and budget utilization. APTS marked a major change from the preceding pharmacy services in a number of ways. In the first place, the program has made renovation and reorganization of the dispensing units. The program rearranges dispensing workflow to enhance prescription evaluation, drug use counseling and patient convenience. The program renovates old dispensaries by opening two doors (entrance and exit doors). Moreover, the APTS program reorganizes the dispensing setup as the prescription evaluator, biller, cashier, and counselor (separate cubicles for prescription evaluator, cashier, and counselor). The program also redefines the responsibilities of dispenser, cashier and pharmacy accountant according to the workflow (Fig 1).

**Fig 2:**
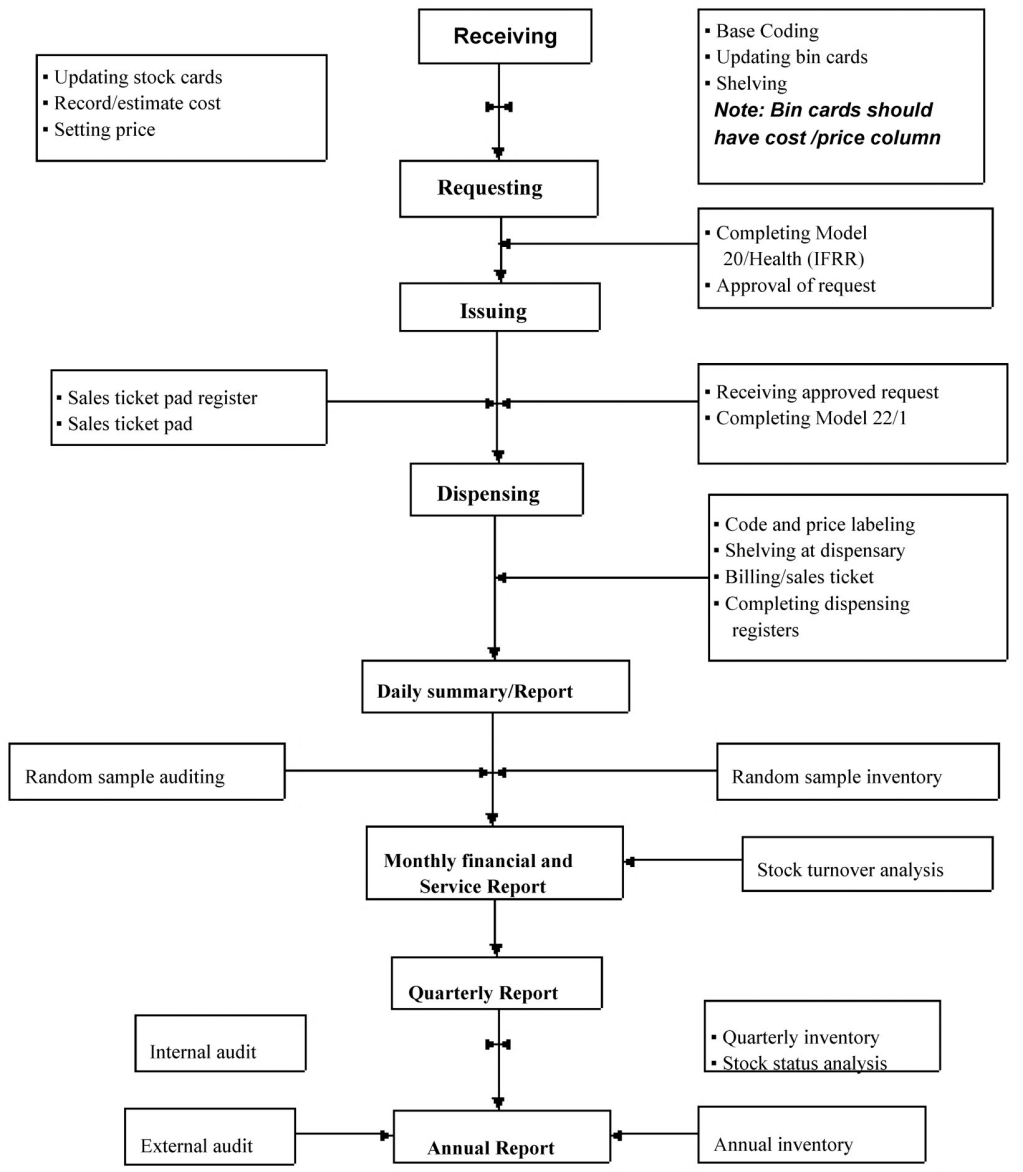
APTS transaction at health facility (Source: APTS training guideline, 2016).

Secondly, the APTS program helps to administer pharmaceutical budget efficiently and visualizes the implementation of health care financing by documenting details descriptions of the pharmaceuticals consumption and by assisting the preparation of the monthly financial and service report. A monthly service report is compiled every month concerning the various types of service information recorded during the month, like the number of drug use counseling, number of patients serviced, availability of medicines and affordability of prices. Thirdly, the program helps to audit pharmaceutical services and transactions at any time. This is because of APTS program has transparent transactions that enable the tracking of information on items with their costs received to the facility, items issued to dispensing outlets, items dispensed to end users and items expired or lost.There are three main types of auditing (financial, product and service auditing) in APTS.The financial auditing includes evaluation of the received items by vouchers with costs, issued items with costs and dispensed items with prices. Service auditing includes; measuring workload and analysis with the APTS standards, the patient served, the number of counseling, the Drug and Therapeutic Problem (DTP) identified, and patients’ knowledge on dispensed medicines. The product auditing includes: checking of products received with expiry dates and batch number, the number of expired medicines with records; the stock on hand, received, issued, dispensed against the ending stock of specific products. Dispensing units are audited every month, whereas the store is audited every three months. Fourthly, the program aid in determining accurate dispensers’ deployment (workload analysis), performance monitoring and on the job training. Fifthly, APTS aids to utilize pharmaceuticals budget efficiently by using methods like price setting both for budget and program pharmaceuticals; generating a daily summary; assigning bin ownership at dispensing units; preparing facility-specific medicine list with products grouped as vital, essential, and desirable; conducting ABC (Always, Better, Control)-VEN (Vital, Essential, Nonessential) matrix analysis to identify the crucial medicines; and performing stock status analysis to identify the usable stock amount against unusable stock amount (Fig 2).

Sixthly, the program aids to assign codes for drugs, supplies, raw chemicals, and medical laboratory reagents. The objective of coding is to ensure the traceability of medicines and transactions at any point. It also makes pharmaceuticals transactions transparent and easily understandable for non-health professionals (auditor and pharmacy accountant) [16].

Evidence from the national assessment indicated that APTS program had the capacity for improving pharmaceutical transactions and services at the secondary and tertiary level health care facilities [15]. However, there is no study that assessed APTS program effect on the medicine prescribing practices, dispensing time, availability of prescribed medications, and affordability of prescribed medicines at primary level health care facilities. Therefore, the aim of the present study was to compare WHO drug use indicators between APTS and Non-APTS primary level hospitals in southern Ethiopia.

## Methods and materials

### Study area and period

Ethiopia administratively is divided into nine regions and two city administrations. Data for the study was collected in the Southern Nation, Nationality and People Republic (SNNPR) region, a predominantly densely populated region. The region is made up of 13 zones, 8 special woredas and one city administration with a total population of 19,170,007 [17]. According to the health transformation plan-I, there are 3874 health posts, 717 health centers and 63 hospitals in the region. The region has six private hospitals, 30 public primary hospitals, 23 public secondary hospitals, and 4 public tertiary hospitals. The research was conducted from January 2018 to December 2018.

### Study design

The study was carried out using a comparative-cross sectional study design, which assessed and compared drug use indicators by using the WHO methodology in primary level hospitals participating in APTS versus Non-Auditable Pharmaceutical Transactions and Services (Non-APTS).

### Sample size determination and sampling technique

#### Health facility

For the purposes of the comparative analytical method, the primary level hospitals found in the southern region were first classified into two categories: APTS and Non-APTS primary level hospitals. According to WHO recommendations, to give reasonable accuracy when making conclusions from observed variations between the intervention and non-intervention comparison groups there should be at least 10 facilities in each category [9]. There are 10 primary hospitals fully implimented APTS, 16 primary hospitals without APTS and 4 primary hospitals at the initial phase of implementing APTS in the region. This study uses data from 20 primary level hospitals (10 with APTS and 10 without APTS). The ten Non-APTS primary hospitals were randomly selected from 16 Non-APTS primary hospitals in the region. All the ten primary hospitals that have been fully implemented the program were included in the study. In our study APTS practicing hospitals were those facilities implementing the program at least for one year. Primary hospitals that failed to complete these inclusion criteria were excluded from the study. The ten selected APTS primary hospitals have been implemented the program for one year. Four primary hospitals with APTS were excluded from the study since they were in the process of implementing the program. Non-APTS primary level hospitals characteristics were comparable to APTS hospitals in their type of practice, patient load, geographical location and ownership type (public or private).

#### For health facility indicators

Thirty essential medicines were chosen from each primary level hospital as per WHO standard which is a minimum of 15 key medicines in each facility [9]. These essential medicines being used for the treatment of the top ten diseases of the respective primary level hospitals were selected by communicating with dispensers, prescribers and reviewing national treatment guidelines.

#### For patient care indicators and complementary indicator (satisfaction)

WHO suggests that a minimum of 100 samples of patients per facility to be used to compare patient related indicators and patient satisfaction indicators between health care facilities [9]. For this study, a total of 1000 samples of respondents were evaluated in APTS primary hospitals and 1000 samples in Non-APTS primary level hospitals. We reviewed last month’s prescription records of the daily flow of patients in the 20 primary level hospitals. The total number of patients who will visit the outpatient pharmacy during the one month study period was computed for each hospital. The number of patients to be surveyed per day during a one-week data collection period was estimated. By dividing the daily patients visit with the number of patients to be interviewed per day to get the interval (K^th^). For instance, in the case of the Non-APTS hospital-1 (N-1), the mean daily patients flow to the outpatient pharmacy was estimated to be about 48. Thus, the total number of patients who will visit the outpatient pharmacy during the one-week data collection period was computed. The number of patients to be interviewed per day during the one week of data collection period was estimated to be 14. By dividing the daily patients visit with the number of patients to be interviewed per day, ever third patients available at the outpatient pharmacy during the one-week data collection period was included by systematic random sampling method. A similar procedure was applied across the remaining primary level hospitals.

#### For prescribing indicators and complementary indicator (average cost of drugs)

According to the WHO standard, minimum of 100 prescribing encounters per health facility should be included in a comparative study to compare prescribing practice between comparison health facilities [9]. Therefore, a total of 1000 prescriptions in APTS hospitals and 1000 in Non-APTS primary hospitals were assessed in the study. In evaluating prescribing indicators, 100 prescriptions per hospital were chosen from among those prescribed between January 2017 and December 2017. The prescriptions written in every quarter were separated and then 25 prescriptions were selected from each quarter using systematic random sampling.

### Inclusion and exclusion criteria

#### Inclusion criteria

Patients who were 18 years or older, who were willing and had their medicines orders filled at the outpatient pharmacy of the hospital were included in the study. Outpatient department prescriptions with at least one or more prescribed drugs were included in the study.

#### Exclusion criteria

Patients who were not physically and mentally capable of being interviewed at the time of data collection were excluded from the study. The prescriptions coming up from consultation in the emergency room were excluded from the study.

### Data collection instrument, measurements, and techniques

The data collection tools initially prepared in English and then translated to the local language (Amharic), and then retranslated into English to ensure the consistency of the tools. The data collection tool consisted of two parts, the first part which focused on the socio-demographic characteristics and health care measures of respondents and the second part on drug use indicators (patient care, prescriber, health facility and complementary). Both core drug use indicators and complementary drug use indicators were evaluated according to the WHO/International Network of Rational Use of Drugs (INRUD) guidelines [9]. Data were collected by using structured questionnaires for prospective study and WHO designed standard-based data collection checklists for a retrospective study.

Twenty-six pharmacy technicians who were not taken APTS training and who were working out of the selected hospitals were selected for data collection. Five clinical pharmacists who were working out of the selected hospitals were recruited for supervision. Three days of training about research ethics and data collection procedures were given for supervisors and data collectors. The data collectors informed the patients in the waiting room about the survey in general terms, obtaining their written consent to participate.

#### Health facility indicators measurement and collection techniques

A standard health facility indicator observation checklist was used to collect data relating to the availability of essential medicines and standard treatment guidelines [9].The WHO health facility indicators were used in this research. The health facility indicators that were measured included:

1. Availability of essential medicines list was assessed to indicate the degree to which essential medicines are available at primary level hospitals.Thirty essential medicines were chosen from each primary level hospital as per WHO standard which is a minimum of 15 key medicines in each facility [9]. The percentage availability of essential medicines was calculated to measure the availability at primary hospitals of essential medicines for the treatment of common diseases. Data collectors were collect data on the availability of essential medicines by visiting each hospital’s medical store.
2. Stockout duration of essential medicines was calculated to measure the historical availability of essential medicines to treat the top ten diseases of the respective primary level hospitals. A retrospective assessment was carried out by reviewing the stock cards of the hospitals covering a period of 12 months. The number of days for which essential medicines were not available within 12 months was recorded in the data collection format. The average stockout period was calculated by dividing the number of days for which essential medicines were not available within 12 months to the review period.

#### Patient care indicators measurement and data collection techniques

Based on the WHO manual on how to evaluate drug use at health facilities outpatient patient care related indicators consist of the mean dispensing time, the mean consultation time, the proportion of drugs actually dispensed to patients, the percentage of medicines sufficiently labeled and patient’s knowledge of the correct dosage. A standard patient care form was used to collect data relating to the dispensing time, consultation time, labeling and number of drugs actually dispensed[9]. The patient care indicators that were measured included:

1. The average consultation time was calculated to determine the time that medical personnel spend with patients during consultation and prescribing. It was computed by dividing the whole time for a consecutive of consultations in minutes to the number of consultations. Observations were made in the consultation room without disrupting the consultation process. Data collectors recorded the time that the medical personnel spend with patients during the consultation and prescribing. A stopwatch was used to measure the consultation time.
2. The average dispensing time was calculated to measure the time between arriving at the outpatient pharmacy encounter and leaving. Hence, it was computed by dividing the total time for dispensing medicines to a sequence of patients in seconds by the number of encounters. Waiting time was not considered. Observations were made in the dispensing room without interrupting the dispensing process. Trained data collectors recorded the time between the patient arriving at the outpatient pharmacy encounter and leaving. A stopwatch was used to measure the dispensing time.
3. The percentage of drugs actually dispensed was calculated to measure the capacity to which primary level hospitals are able to dispense the medications which were ordered. It was calculated by dividing the total number of medicines actually dispensed at the primary hospitals to the whole number of medicines prescribed and multiplied by 100%. Data on the number of medicines prescribed were recorded from prescription. The number of drugs dispensed collected by examining the patient has really received.
4. The percentage of drugs adequately labeled was calculated to determine the degree to which pharmacists write crucial drug information on the drug packages they dispense. The percentage was calculated by dividing the number of medicine packages consisting of at least the patient name, medicine name, and frequency of administration to the total number of medicine packages and multiplied by 100%. Data on the labeling of drugs were collected by observing drug information on the drug packages that were dispensed to patients.
5. The proportion of patients’ knowledge of the right dosage was computed to determine the effectiveness of the counseling given to patients on the dosage frequency of the medicines they obtain. The percentage was computed by dividing the number of patients who can correctly recall the dosage frequency for entire medicines to the whole number of patients interviewed and multiplied by 100%. Data collectors interviewed patients as they exited the outpatient pharmacy, focusing on knowledge about dispensed medications (name, dosage, and frequency of administration, route of administration, drug interactions, possible adverse effects and attitude toward one or higher medicine dose missed). Responses to knowledge items were transcribed and compared to the prescription. Information not stated in the prescription (indication, attitude when dosages are missed, side effects and drug interactions) were based on a national drug formulary. For each item, if respondents gave a right response, they scored 1 point; if incorrect response (no or do not know) was given, the score for the items was 0. The name of the drug was considered right when pronounced exactly. The dosage was considered right when there was a similarity between the patient response and the amount to be administered at each time. Frequency, forms of use and duration of treatment (concord between the patient response and the acute or chronic nature of prescribed therapy) were evaluated in the prescription.

In addition, basic socio-demographic and health-related information was collected such as age, sex, residence, religion, marital status, occupational status, educational status, payment status, health status, service sought for and the number of visits.

#### Prescribing indicators measurement

Based on WHO medicine use investigation manual, prescribing indicators consist the mean number of drugs ordered per encounter, the proportion of medicines ordered by nonproperty name, the proportion of prescriptions with antibiotic, the proportion of encounters with injections and proportion of ordered medicines from key medicine list. A standard prescribing indicator form was used to collect data relating to the prescribing indicators [9]. All indicators are explained below.

1. The mean number of medicines ordered per prescription was used to measure the level of polypharmacy. Therefore, the average number of medicines was computed by dividing the whole number of medicines ordered to the number of prescriptions evaluated. A fixed-dose combination (FDC) of medicines ordered for a single disease was considered as a single medicine.
2. The proportion of medicines ordered by nonproperty name was used to measure the extent of prescribing by nonproperty name. It was computed by dividing the number of medicines ordered by nonproperty names to the whole number of medicines prescribed and multiplied by one hundred percent.
3. The proportion of prescriptions in which an antibiotic was ordered was used to determine the general use of expensive and excessively used forms of treatment. The percentage was computed by dividing the number of encounters during which an antibiotic was ordered by the whole number of encounters evaluated and multiplied by one hundred percent.
4. The proportion of prescriptions with an injection ordered was computed to determine costly forms of medicine therapy and expensive forms of drug therapy. The percentage was computed by dividing the number of encounters during which an injection was ordered by the whole number of encounters evaluated and multiplied by one hundred percent.
5. The proportion of medicines ordered from key medicine list was used to determine the extent to which prescribing performance adherence to a state medicine policy. It was computed by dividing the number of medicines ordered which are in key medicine list by the whole quantity of medicines ordered and multiplied by one hundred percent.

Data about prescribing indicators were taken from systematically sampled prescription registrations retrospectively and filled in prescribing indicator form.

#### Complementary indicators

The complementary indicators that were measured include patients’ satisfaction with the pharmacy service they received and the average drug cost per encounter. The data on the level of satisfaction of patients with the services of the outpatient department (OPD) dispensing unit in APTS and Non-APTS primary hospitals was done using a structured questionnaire which was adopted from 5 previously applied instruments [18–20]. The questionnaire was used to assess patients’ satisfaction with dispensing time, waiting time, cost of medicines, availability of medicine, the professionalism of dispenser, privacy during dispensing, cleanness of pharmacy, space of pharmacy, drug information, the respect shown by professionals and quality of medicine level. The responses were offered using a 5 mark Likert scale (1-very dissatisfied, 2-dissatisfied, 3-not sure, 4-satisfied and 5-very satisfied). The satisfaction level data were collected by pharmacy technicians through interviewing patients after they had their prescriptions filled at the OPD pharmacy. The average drug cost per encounter was assessed to measure the cost of drug treatment. The average cost was calculated by dividing the total cost of all drugs prescribed to the number of encounters surveyed. Data about the cost of prescribed drugs were extracted retrospectively from sampled prescription records. As a measure of medicine affordability, the number of day’s wages required to pay for the average cost of medicines per encounter was computed from the daily wage of the lowest-paid unskilled governmental worker in Ethiopia. (40 Ethiopian Birr/day = 1.43 United States Dollar).

### Statistical analysis

We used Epi Data Version 3.1.software for data entry. After that, the data was exported and analyzed using Statistical Packages for Social Sciences (SPSS) version 20. The mean and standard deviations were calculated for parametric continuous variables. All the categorical variables were examined using the chi-square test (χ^2^). We compared the respondents’ socio-demographic characteristics, health care measures, percentage of drugs adequately labeled, percentage of patients with adequate knowledge of dosage, percentage of encounter with an antibiotic, percentage of encounter with an injection, encounters with only one drug prescribed and encounter with five or more drugs prescribed between APTS and Non-APTS primary level hospitals using Chi-squared tests. The differences in mean scores of consultation time, dispensing time and the number of drugs per prescription between the 2 primary level hospitals were examined by independent-2-sample t-tests. Bi-variate binary logistic regression analysis was employed to compare knowledge of patients on dispensed medicines between APTS and Non-APTS primary hospitals, while multivariable binary logistic regression analysis was then employed to compare knowledge on medicines between the two primary hospitals by controlling for respondents’ age, sex, religion, residence, payment status,self-reported health status, service sought for, marital status, number of visits, employment status and educational status.

Mann-Whitney U test was used to test for the difference between APTS and Non-APTS primary level hospitals with each satisfaction dimension Likert scores and data were presented as the mean rank and a *p*-value. Multiple linear regressions were used to compare the total satisfaction score between the two primary level hospitals after controlling for all respondents’ socio-demographic characteristics (age, sex, residence, religion, marital status, employment status, educational status) and health care measures (self-reported health status, service sought for, payment status, number of visits). The results were expressed as odd ratio with 95% confidence interval (95%CI). For all tests performed in the research, a P value of less than 0.05 was taken as the statistically significant level.

### Ethical consideration

Ethical approval for the research was obtained from the Institutional Ethics Review Board (IRB) of Arbaminch College of Health Sciences. The authorized permission letter was obtained from selected areas health office and the data collection started after cooperation letter was written to all twenty primary level hospitals. Participant informed written consent and assent was obtained and the respondents were guaranteed of confidentiality.

## Results

### Characteristics of study participants

A total of 2000 patients (1000 from APTS and 1000 from Non-APTS hospitals) were interviewed with a 100% response rate. In both facilities, the majority of respondents tended to be males (52.9% in Non-APTS and 52.0% in APTS primary hospitals), were rural dwellers (53.6% in Non-APTS and 54.7% in APTS),were married (76.7% in Non-APTS and 76.2% in APTS), were Christians (57.6% and 59.1%), were farmers (40.5% in APTS and 44.2% in Non-APTS), were not able to read and write (31.7% in APTS and 32.3% in Non-APTS), paid for medications (51.8% in APTS and 55.8% in Non-APTS), had more than three visits in the last 12 months (57.6% in APTS and 59.1% in Non-APTS) and taking medications for themselves (51.8% in APTS and 55.8% in Non-APTS). There was no significant difference between APTS and Non-APTS primary hospitals in the sociodemographic and health related characteristics of respondents (Table 1). There was no significant difference between APTS and Non-APTS facilities in the self-reported health status, payment status, number of visits, service sought for and sociodemographic characteristics of respondents (Table 2).

**Table 1:** Comparison of the socio-demographic characteristics and health care measures between study population of APTS and Non-APTS primary hospitals.

Variables	Overall n (%)	APTS hospitals n(%)	Non-APTS hospitals n(%)	*P*- value
Age category				
18–27	556(27.8)	289(28.9)	267(26.7)	0.599
28–37	562(28.1)	282(28.2)	280(28.0)	
38–47	390(19.5)	194(19.4)	196(19.6)	
≥48	492(24.6)	235(23.5)	257(25.7)	
Sex				
Male	1049(52.4)	520(52.0)	529(52.9)	0.720
Female	951(47.6)	480(48.0)	471(47.1)	
Residence				
Urban	917(45.9)	453(45.3)	464(46.4)	0.654
Rural	1083(54.1)	547(54.7)	536(53.6)	
Religion				
Christian(Orthodox)	1167(58.3)	576(57.6)	591(59.1)	0.482
Protestant	462(23.1)	228(22.8)	234(23.4)	
Muslim	371(18.6)	196(19.6)	175(17.5)	
Marital status				
Married	1529(76.4)	762(76.2)	767(76.7)	0.833
Unmarried	471(23.6)	238(23.8)	233(23.3)	
Occupational status				
Government employee	463(23.2)	241(24.1)	222(22.2)	0.426
Private employee	136(6.8)	67(6.7)	69(6.9)	
Farmer	847(42.3)	405(40.5)	442(44.2)	
Merchant	146(7.3)	80(8.0)	66(6.6)	
No job	408(20.4)	207(20.7)	201(20.1)	
Educational status of respondents				
Not able to read and write	640(32.0)	317(31.7)	323(32.3)	0.197
Primary school	601(30.1)	294(29.4)	307(30.7)	
Secondary school	401(20.0)	192(19.2)	209(20.9)	

**Table 2:** Comparison of the socio-demographic characteristics and health care measures between study population of APTS and Non-APTS primary hospitals (continue).

Variables	Overall n (%)	APTS hospitals n(%)	Non-APTS hospitals n(%)	P- value
Higher education	358(17.9)	197(19.7)	161(16.1)	
Payment status				
Free	124(6.2)	69(6.9)	55(5.5)	0.228
Cash/credit	1876(93.8)	931(93.1)	945(94.5)	
Self-reported health status				
Good	1069(53.4)	547(54.7)	522(52.2)	0.282
Bad	931(46.6)	453(45.3)	478(47.8)	
Service sought for				
Self	1076(53.8)	518(51.8)	558(55.8)	0.080
Other	924(46.2)	482(48.2)	442(44.2)	
Number of visit				
First visit	418(20.9)	220(22.0)	198(19.8)	0.480
Second visit	415(20.7)	204(20.4)	211(21.1)	
Follow up	1167(58.4)	576(57.6)	591(59.1)	

#### Health facility-specific indicators

**Fig 3:**
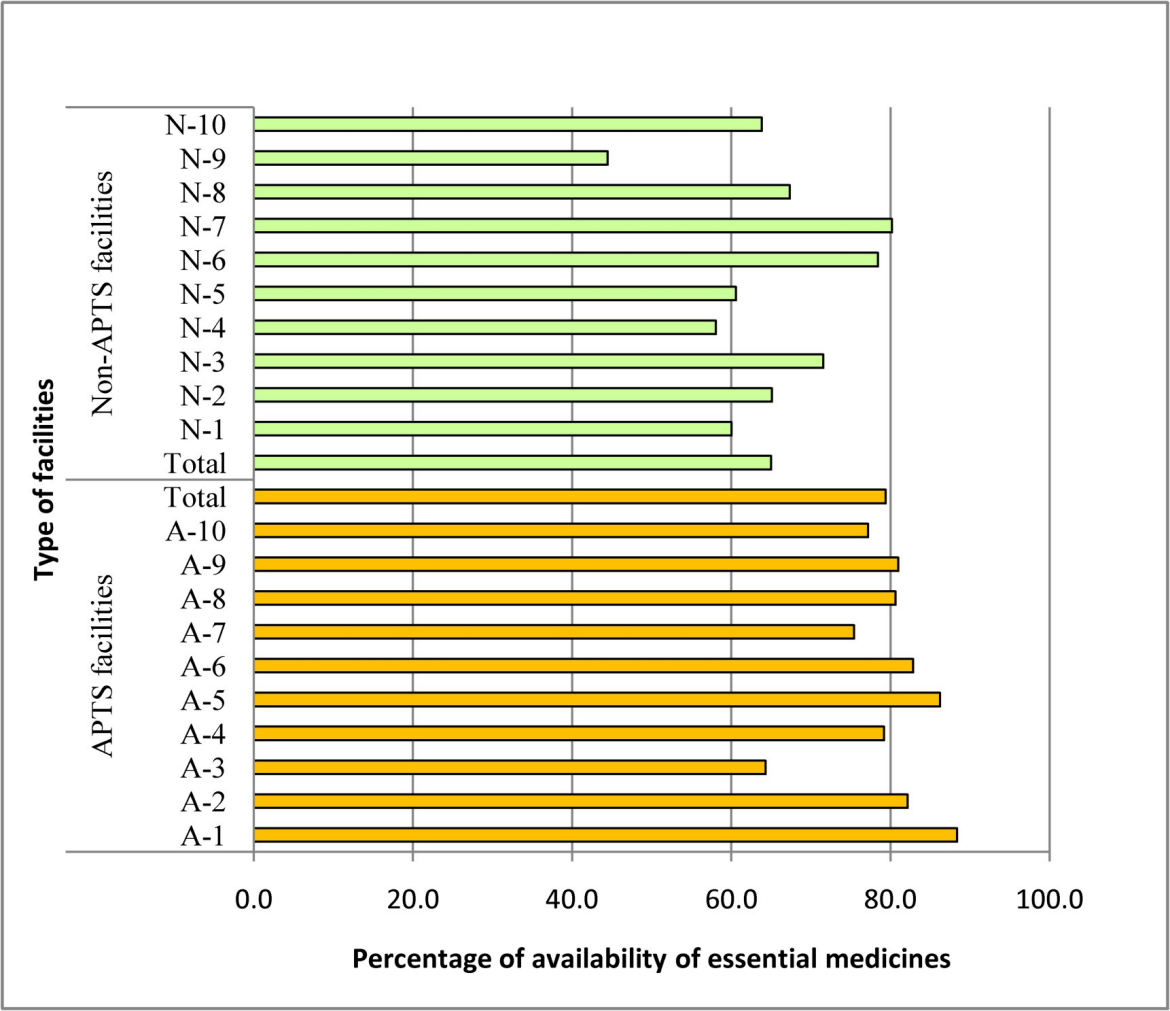
Percentage of availability of essential medicines between facilities participating in APTS and Non-APTS program.

The percentage availability of the essential drug list document in Non-APTS facilities was 80% while in APTS facilities, it was 100%. The percentage availability of essential drugs in APTS facilities was 79.4% (64.3–88.4) while in Non-APTS facilities was only 65% (60–80.2) (Fig 3). Our results showed that the percentage availability of essential medicines in APTS facilities was statistically higher than Non-APTS facilities (U = 13, p = 0.005). On average, selected essential drugs were stock out for 63 days/year in Non-APTS facilities whereas in APTS facilities drugs were stock out for 38 days/year.

#### Patient care indicators

**Fig 4:**
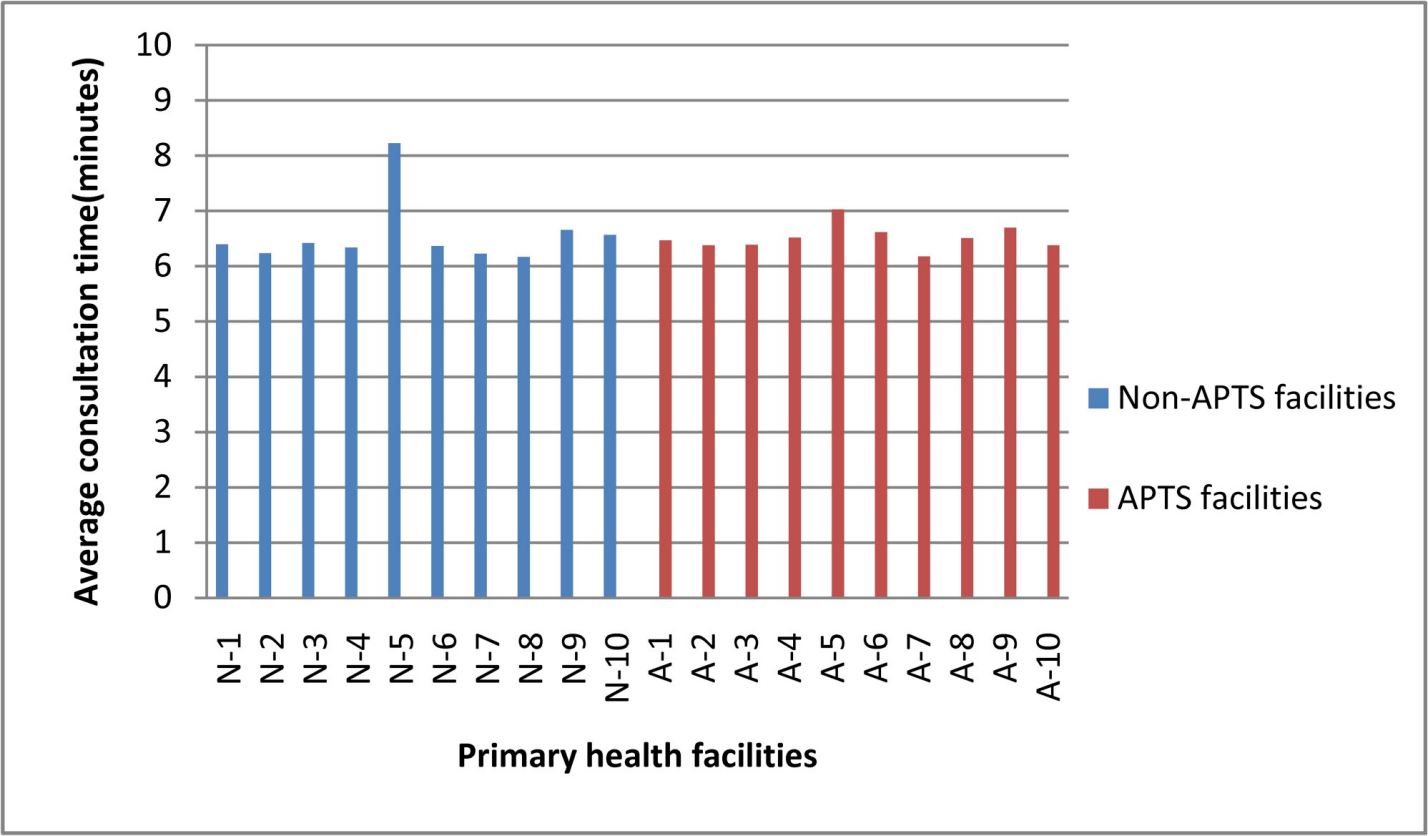
Average consultation time in minutes between individual APTS and non-APTS facilities. N-Non-APTS A-APTS.

**Fig 5:**
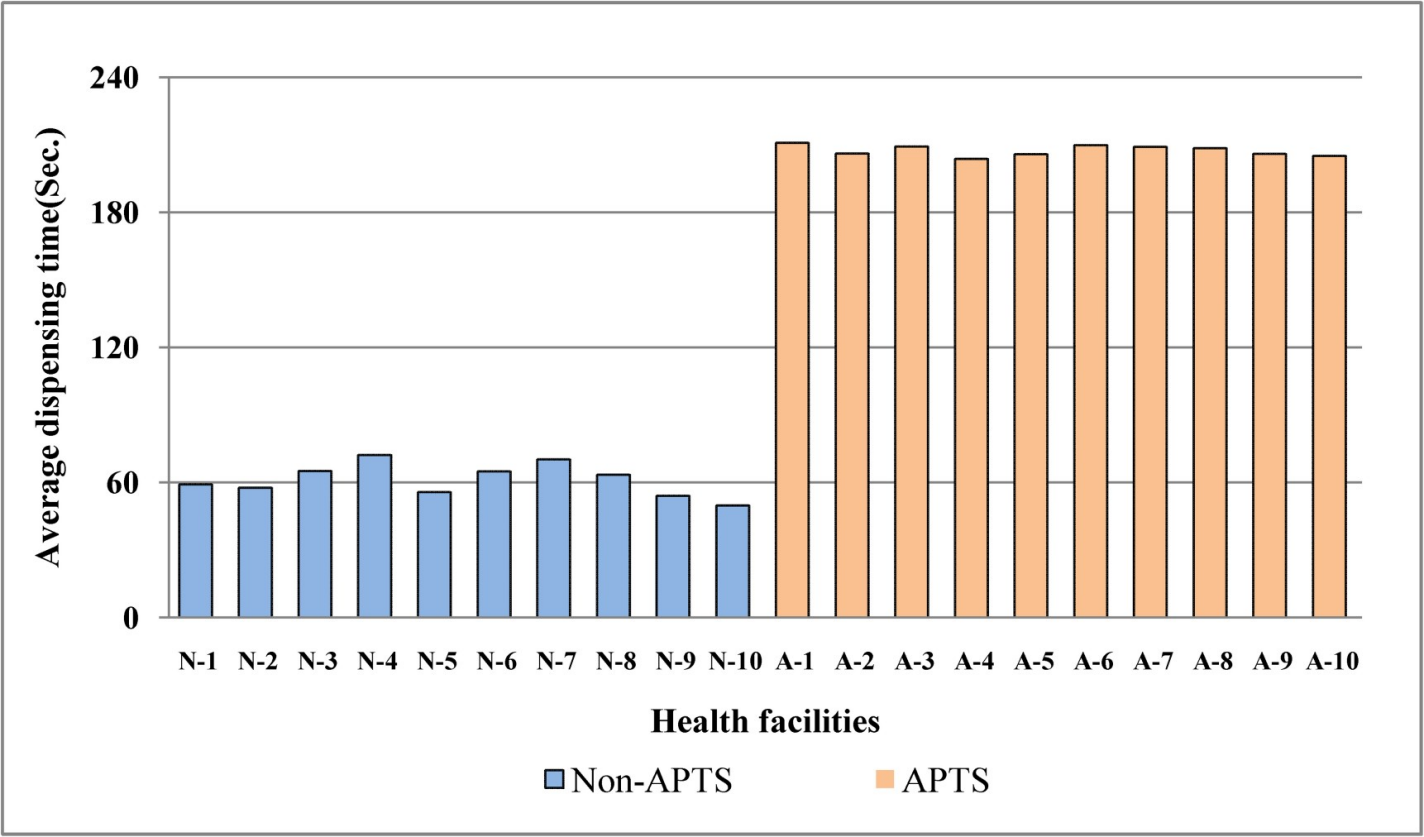
Average dispensing time in seconds between APTS and non-APTS facilities. N: Non-APTS facilities, A: APTS facilities.

**Fig 6:**
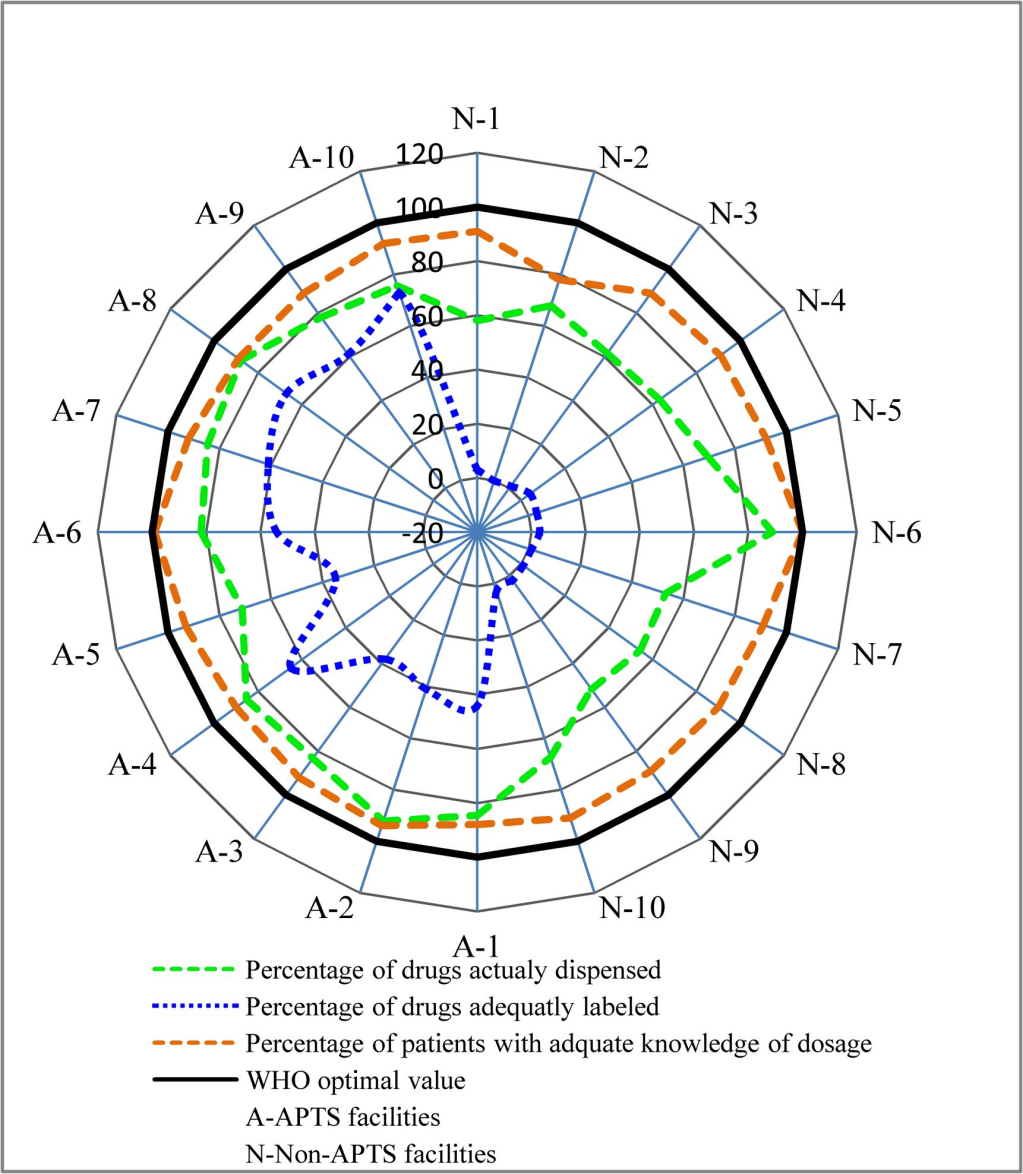
Percentage of drug actually dispensed drugs, drugs adequately labeled and patients with adequate knowledge by each facility of APTS and non-APTS. N: Non-APTS facilities, A: APTS facilities.

The mean consultation time and dispensing time in APTS facilities were found to be 6.5 minutes and 3.46 minutes, respectively. In Non-APTS facilities, the average consultation and dispensing time were 6.6 minutes and 1.02 minutes, respectively (Table 2). The lowest average consultation and dispensing time were seen in Non-APTS facility (6.2 and 0.83 minutes), respectively (Figs 4 and 5). The average consultation time was generally below the WHO ideal value for both groups of facilities. The proportion of drugs actually dispensed was 97.59% in APTS facilities and 76.44% in Non-APTS facilities with the lowest value seen in Non-APTS facility (51.65%). Only 53.9% of dispensed prescriptions at APTS facilities were adequately labeled.Among dispensed prescriptions within the Non-APTS primary facilities, only 2.2% of them were sufficiently labeled when taken together with zero percentage documented in Non-APTS facilities (Fig 6). In addition, 91.60% and 90.47% of respondents knew about the dosage of their dispensed medication in APTS and Non-APTS facilities. The comparison of WHO patient care indicators between APTS and Non-APTS facilities related to the average dispensing time, percentage of drugs actually dispensed and percentage of drugs adequately labeled, showed highly significant differences (p<0.001) (Table 3).

**Table 3:** Comparison of WHO patient care indicators (consultation time, dispensing time, labeled medicines and actually dispensed) between encounters of APTS primary hospitals and Non-APTS primary hospitals.

Patient-Care Indicators	APTS facilities	Non-APTS facilities	WHO ideal value	p-value
Average consultation time(minutes) ±SD	6.5±1.73	6.6±1.74	≥10minutes	0.519
Average dispensing time(minutes) ±SD	3.46±0.25	1.02±0.29	≥3minutes	0.000
Percentage of drugs actually dispensed	97.59±11.54	76.44±35.22	100	0.000
Percentage of drugs adequately labeled	53.9	2.1	100	0.000
Percentage of patients with adequate knowledge of dosage	91.8	90.2	100	0.241

More than 77.3% of APTS respondents knew that the name of the drug while only 22.7% of Non-APTS respondents correctly identified the name of the dispensed medications. The dosage was recalled in 916 (91.6%), time of administration in 918 (91.8%), route of administration in 962 (96.2%), treatment duration in 932 (93.2%), possible side effects in 759 (75.9%), possible drug interactions in 671 (67.1%), storage condition in 825 (82.5%), action during missed dose in 455 (45.5%) of the respondents in APTS. The extent of participant’s knowledge on treatment duration, possible side effects, possible drug interaction, drug storage condition, and action for a missed dose of the drugs showed that Non-APTS respondents had less knowledge on dispensed drugs than APTS respondents.

The possible side effects, interactions, storage condition, and missed dose were the most lacking pieces of information in Non-APTS facilities. There was statistically significant difference between the two facilities in relation to respondents in recalling treatment duration, possible side effects, possible drug interactions, storage condition and action for a missed dose of the drugs dispensed (p<0.001). However, there was no significant difference in recalling dosage, time of administration and route of administration between the two facilities (Table 4).

**Table 4:** Comparison of patients’ knowledge on prescribed medicines in APTS and Non-APTS primary level hospitals: Univariable and Multivariable Logistic Regressions.

Knowledge of medicine items	Correct response		OR^1^ (95% CI)	OR^2^ (95% CI)
	Yes (%)	No (%)		
Name of drug Non-APTS APTS	496(49.6) 773(77.3)	504(50.4) 227(22.7)	1 3.46(2.85–4.20)	1 3.49(2.87–4.25) *
Dosage Non-APTS APTS	907(90.7) 916(91.6)	93(9.3) 84(8.4)	1 1.12(0.82–1.52)	1 1.12(0.82–1.54)
Route of drug administration Non-APTS APTS	948(94.8) 962(96.2)	52(5.2) 38(3.8)	1 1.39(0.91–2.13)	1 1.40(0.90–2.17)
Time of administration Non-APTS APTS	902(90.2) 918(91.8)	98(9.8) 82(8.2)	1 1.22(0.89–1.65)	1 1.21(0.89–1.65)
Possible side effects Non-APTS APTS	99(9.9) 759(75.9)	901(90.1) 241(24.1)	1 28.66(22.25–36.92)	1 30.20(23.30–39.14) *
Possible drug interactions Non-APTS APTS	142(14.2) 671(67.1)	858(85.8) 329(32.9)	1 12.32(9.89–15.37)	1 13.10(10.43–16.43)*
Storage condition of medicine Non-APTS APTS	387(38.7) 825(82.5)	613(61.3) 175(17.5)	1 7.45(6.07–9.18)	1 7.57(6.14–9.34) *
Treatment duration Non-APTS APTS	710(71.0) 932(93.2)	290(29.0) 68(6.8)	1 5.60(4.22–7.42)	1 5.83(4.38–7.75) *
Missed dose Non-APTS APTS	160(16.0) 455(45.5)	840(84.0) 545(54.5)	1 4.38(3.55–5.41)	1 4.52(3.65–5.59)*

*P value significant at 0.05

CI-Confidence Interval

OR^1^- Odds ratio for unvariable logistic regression

OR^2^-Adjusted program effect (95%CI) The program effect was examined using multivariable logistic regression models, where the dependent variables are each items knowledge, while independent variables are sex, marital status, residence, self reported health status, religion,occupational status, level of education, number of visit, age, payment status and service sought for.

#### Prescriber indicator

For the measurement of prescribing indicators, a total of 2000 prescription encounters (1000 from APTS and 1000 from Non-APTS hospitals) were included in the assessment making 100% completion rates. For the evaluation of prescribing indicators, a total of 2000 prescription encounters (1000 from APTS and 1000 from Non-APTS primary hospitals) were incorporated into the study. The average number of drugs per prescription was 2.32 (±1.26) and 2.84 (±1.17) in APTS facilities and Non-APTS facilities respectively. From total prescriptions assessed in APTS facilities, 31.6% contained only one drug per prescription and 6.9% contained five or more drugs per prescription. On the other way from total prescriptions investigated in Non-APTS facilities, 7.9% contained only one drug per prescription and 10.7% contained five or more drugs per prescription. Amongst the prescription evaluated, the study shows that 97.6% and 98.6% of drugs prescribed by generic name in Non-APTS and APTS facilities, respectively).

**Fig 7:**
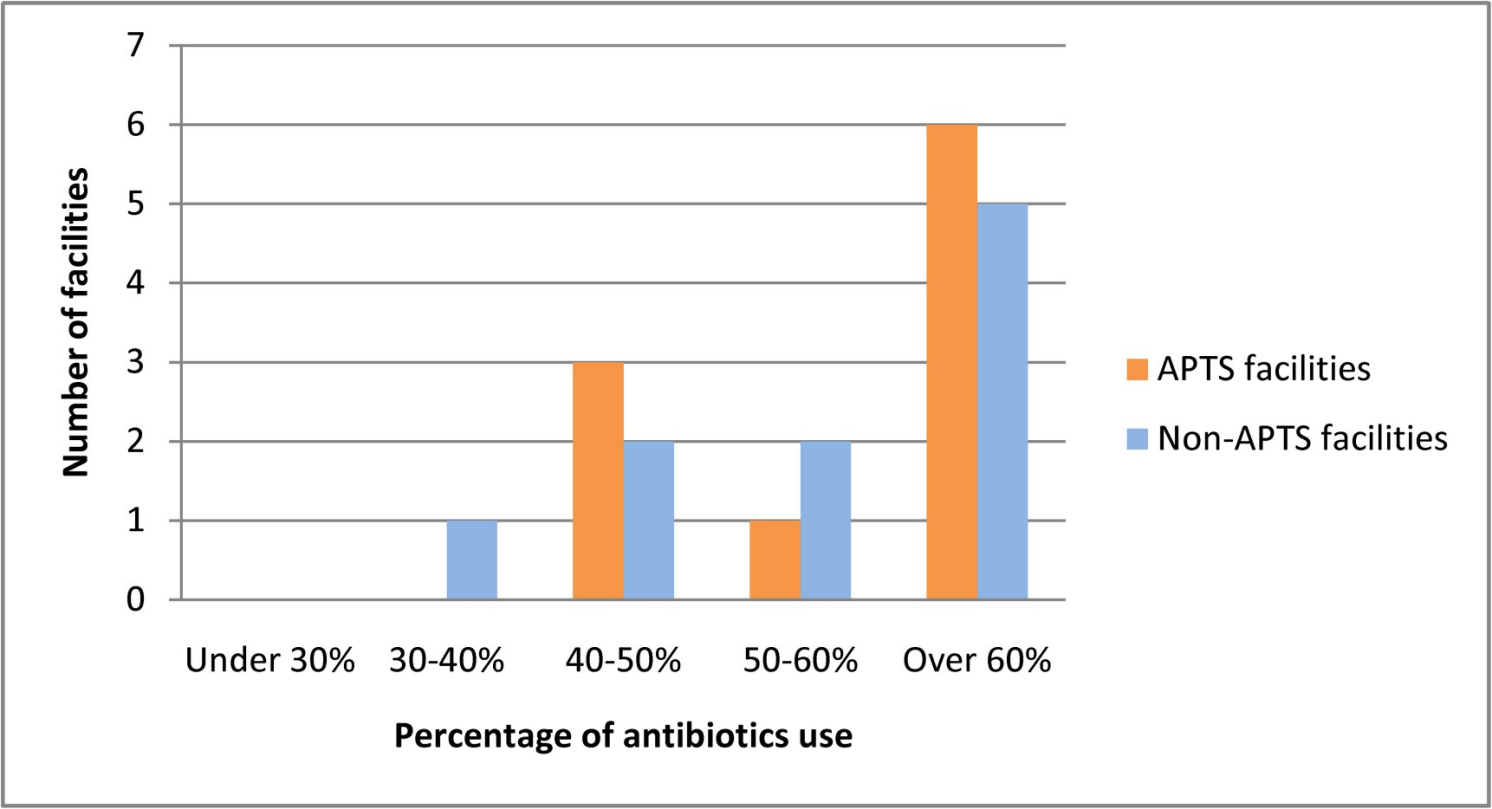
Percentage of antibiotics use between APTS and Non-APTS facilities.

The percentage of encounters with antibiotics was 57.8% in APTS facilities and 56% in Non-APTS facilities (Table 4). The major types of antibiotics were Ceftriaxone (0.5g injection) and Sulphamethoxazole + Trimethoprim (800mg +160mg tablet) at APTS facilities, while they were Amoxicillin (500mg capsule) and Cloxacillin sodium (500mg capsule) at Non-APTS facilities. In both sites, 60% of the prescriptions contained antibiotics in more than 50% of the facilities (Fig 7). Our results show that the percentage of encounters with injection was 38.2% in APTS facilities and 40.5% in Non-APTS facilities. The mean percentage of medicines prescribed from the facility formulary or national essential drug list was 96.2% in APTS facilities and 93.6% in Non-APTS facilities. Between comparing primary facilities with and without APTS, the percentage of prescriptions contained at least one injection drug and the percentage of prescriptions consisted of antibiotics was similar between facilities (p>0.05). However, there was significant difference in the average number of drugs per encounter, the average number of drugs prescribed by generic name and percentage of drugs prescribed from formulary between the two facilities (p<0.05) (Table 5).

**Table 5:** Comparison of the WHO prescribing indicators between encounters/ drugs of APTS and Non-APTS facilities.

WHO prescribing indicators	APTS facilities	Non-APTS facilities	WHO standards	p-value
Number of drugs per encounter (mean ± SD)	2.32±1.26	2.84±1.17	(1.6–1.8)	*P*<0.001^†^
Encounters with only one drug prescribed	31.6%	7.9%		*P*<0.001*
Encounter with two upto four drugs	61.5%	81.4%		
Encounter with five or more drugs prescribed (polypharmacy)	6.9%	10.7%		
Percentage of drugs prescribed by generic name	98.60%	97.7%	100	0.040^†^
Percentage of encounter with an antibiotic	57.80%	56.0%	<30(20–26.8)	0.443*
Percentage of encounter with an injection	38.2%	40.5%	(13.4–21.18)	0.314*
Percentage of drugs prescribed from formulary	96.2%	93.6%	100	0.002^†^

SD = standard deviation.

^†^‘t’ test used to compare means.

*chi-square test used to compare frequencies.

### Complementary indicators

#### Patient satisfaction with the pharmacy services they received

The level of satisfaction amongst patients in the APTS facility on the convenience of pharmacy location reference to other services was significantly higher than the patients in Non-APTS facilities (U = 409785.00, p<0.001).The patients in Non-APTS facilities were less satisfied than in APTS about the information for the location of pharmacy (U = 84999.00, p<0.001) and cleanliness of outpatient pharmacy (U = 464553.50, p<0.001). The level of satisfaction amongst respondents in the APTS primary facility on the amount of time they spend waiting for their prescription to be filled statistically significantly higher than the respondents in Non-APTS primary facilities (U = 67513.50, p<0.001). Besides, the level of satisfaction amongst respondents in the APTS primary facilities on the space of dispensary room was statistically significantly higher than the respondents in Non-APTS primary facilities (U = 189782.00, p<0.001). Satisfaction levels related to the performance of attending dispenser were found higher for APTS facilities in most areas of concern. The patients were found to be more satisfied in APTS than in Non-APTS about the clarification of all possible side effects or adverse events (U = 92319.50, p<0.001), dispenser’s explanation about how to take medication (U = 339830.50, p<0.001), time spend on counseling (U = 118205.00, p<0.001), adequately answered questions (U = 424939.50, p<0.001) and information on contraindications (U = 107299.00, p<0.001). Moreover, results revealed that privacy during counseling in APTS facilities was significantly higher than in Non-APTS facilities (U = 73329.00, p<0.001).

The patients were found to be more satisfied in APTS than in Non-APTS regarding the availability of prescribed medicines (U = 356307.00, p<0.001), the label on the drug (U = 318197.50, p<0.001) and quality of the drug dispensed (U = 464660.00, p<0.01).There were no significant differences in the satisfaction level of respect shown by the dispenser, fairness of cost of drug, dispensers’ professionalism and information about storage condition between the two facilities (Table 6).

**Table 6:** Patient satisfaction score between facilities participating in APTS and Non-APTS.

Questions/Dimension of pharmacy service	APTS facilities	Non-APTS facilities	Mann-Whitney U Test	
	Mean Ranks	Mean Ranks	U-Test	*p*-value
Convince of pharmacy location reference to other services	1415.50	585.50	84999.00	0.000
Information for location of pharmacy	1090.72	910.29	409785.00	0.000
Cleanliness of outpatient pharmacy	1035.95	965.65	464553.50	0.000
The space of the dispensary	1310.72	690.28	189782.00	0.000
Satisfaction with the waiting time	1432.99	568.01	67513.50	0.000
Information on how to take your medication	1160.67	840.33	339830.50	0.000
Clarification of all possible side effects or adverse events	1408.18	592.82	92319.50	0.000
Information on contraindications	1393.20	607.80	107299.00	0.000
Information on storage of medicines	1013.36	987.64	487138.50	0.294
Labeling information on dispensed drugs	1182.30	818.70	318197.50	0.000
Availability of prescribed medicines	1144.19	856.81	356307.00	0.000
Fairness of cost of drugs in the outpatient pharmacy	1010.07	990.93	490429.00	0.440
Your feelings of the quality of drug dispensed to you	1035.84	965.16	464660.00	0.003
Respect shown by the dispensers	1007.03	993.97	493467.00	0.564
Privacy during counseling	1427.17	573.83	73329.00	0.000
The dispenser’s professional relationship	1005.74	995.26	494755.50	0.643
Satisfaction with response from staff	1075.56	925.44	424939.50	0.000
The amount of time the dispenser spends with you	1382.30	618.71	118205.00	0.000

Respondents from APTS hospitals were significantly more satisfied than those of Non-APTS primary hospitals [β(CI) = 20.3,95%CI (19.7,28.8); p<0.001] even when adjusted for sex, marital status, self-reported health status, religion, occupational status, level of education, number of visits, age, payment status, and service sought for (Table 7).

**Table 7:** Total satisfaction score reported by participants in APTS and Non-APTS primary hospitals.

Item	APTS Mean(SD)	Non-APTS Mean(SD)	Unadjusted program effect Beta (95% CI)^†^	Adjusted program effect Beta (95% CI)*
Total satisfaction score	68.4(5.22)	47.9(7.34)	20.4(19.87–20.98)	20.3(19.7–28.8) *P*<0.001

^**†**^**-**Examined by independent two sample t-test

*-The program effect was examined by using multiple linear regression, where the dependent variable is total satisfaction score while independent variables are age, sex, marital status, residence, religion, educational level, occupational status,self-reported health status, number of visits, payment status and service sought for (adjusted effect of the program). Beta is calculated with Non-APTS as reference. CI-Confidence Interval.

#### The average drug cost per encounter

The average drug cost per encounter ranged from 17.2–27.84 Ethiopian Birrs (ETBs) across the APTS facilities, with a mean of 19.8ETB for the 10 APTS facilities. The mean cost of drugs per encounter ranged from 22.1–56.4 ETBs across the Non-APTS facilities, with a mean of 45.63 ETBs for the 10 APTS facilities.

## Discussion

To our understanding, the study of FMOH [15] is the only other study comparing drug use among APTS and Non-APTS health facilities. This first time that comparing WHO drug use indicators among primary hospitals with and without APTS program, and it also evidences to extend its implementation to the low-level health tier system of the country like health centers and health posts.

### Health facility specific indicators

The study revealed that all primary hospitals with APTS had a copy of EDL (100%) which is in line with the proposed WHO standard. This result is similar to the reports from Saudi Arabia (100%) [21] and Pakistan (100%) [22].This similarity might be due to the presence of functional drug and therapeutics committee (DTC) in the facilities. However, the percentage availability of a copy of EDL was 80% (optimal value of 100%) in non-APTS facilities. The presence of EDL in APTS hospitals provides numerous benefits like: identify approved drugs for the prescriber; the prescriber will develop a better practice with fewer drugs, drug treatment at a lower cost and continuous supply of drugs. WHO recommends adherence of prescribers to the medicine listed in the EDL when ordering drugs in order to make proper health care for all [9]. Our finding revealed that there was a significant difference in the percentage availability of essential medicines between APTS and non-APTS primary hospitals. The highest percentage was recorded in APTS hospital was 88.4% of the essential medicines were available in stock.This may be due to regular auditing of dispensing units and bin ownership for each essential drug in APTS hospitals [16]. These results are different from a study conducted at a national level that showed that there was no statistically significant difference in the availability of key medicines between APTS and Non-APTS referral hospitals [15]. This difference might be due to the fact that hospital-level variation of the study areas. The absence of essential drugs was a strong sign of poor inventory management in the facility and collapse the health services.

### Patient care indicators

The mean consultation duration was 6.5 min in APTS and 6.6 min Non-APTS facilities. Almost all facilities score less than the ideal WHO standard (10minutes). This implies that the APTS program is not associated with improving the consultation duration. The short consultation time will negatively influence information provision about treatment choices. This short communication could lead to decreased patient satisfaction level and poor treatment effect. Similarly, with regard to the average consultation time, studies from eastern Ethiopia (5.1min) [23], northwest Ethiopia (2.9min) [24], Malawi (2.5min) [25], Saudi Arabia (7.3min) [21], Kuwait (2.8min) [26] and Indonesia (3.0min) [27] showed lower values. However, better consultation durations were observed in Nigeria (11.3min) [28], China (9.5min) [29] and Sweden (22.5min) [30]. Based on our findings, the mean dispensing time in dispensaries of APTS facilities was 3.46 minutes compared to 1.02 minutes in Non-APTS facilities. The average dispensing time in all Non-APTS facilities was lower than the WHO standard (3 minutes). The difference between the two facilities was statistically significant (p<0.001). This could be APTS hospitals were provide medicine counseling service under dispensing counter and pharmacy professionals were assigned to perform specific jobs like counseling or prescription evaluation. The current finding in APTS facilities was better than studies conducted in China (0.42min) [31], Bangladesh (0.38min) [32], Pakistan (0.63min) [22], Saudi Arabia (1.66min) [21], Jordan (0.48min) [33], Brazil (0.28min) [34], Nigeria (0.21min) [35], Swaziland (0.30min) [36] and Mozambique (0.6min) [37]. Very short dispensing time is inadequate for proper labeling and to give effective drug information about the name of drugs, route of administration, dosage, frequency of administration, possible drug interactions, possible side effects, and storage condition of dispensed drugs. Inadequate counseling onmedicines could lead to non-adherence and consequent undesirable effects.

We found that the percentage of medicines actually dispensed was 97.6% and 76.4% in APTS and non-APTS health care facilities respectively which is below the ideal WHO standard of 100%. This percentage indicated that patients were prone to unnecessary medicine charge by private medicine retail outlets where their margin of profit might reach more than one hundred percent. The difference between the two primary level hospitals was statistically significant (p<0.001). We suspect bin ownership and adherence of prescribers to EDL in APTS hospitals contributes to this result. In APTS hospitals, bins are allocated to a specific dispenser at a time. The bin owner has the responsibility to follows the expiry date of medicines, follow the movement of stocks and keep up-to-date information on the stock status of medicines in each bin assigned to him/her. This study revealed that the percentage of medicines actually dispensed in APTS hospitals was higher than reported in eastern Ethiopia (86.2%) [23], Tanzania (56.2%) [38], Jordan (81.8%) [33] and Brazil (66%) [34].

To our understanding, this is the first study that revealed knowledge on possible side effects, possible interaction, missed doses and storage of dispensed medicines is higher for patients served in APTS facility compared with patients in Non-APTS facilities. This can be enlightened by the fact that APTS facility dispensing units have windows with counters and a separate area for drug counseling. According to our results, more than 90% (optimal value 100%) of patients were able to recall the right dosage time of the medicines they received which was higher when compared to other studies done in national level which were 85.4% [15] and eastern Ethiopia 69.8% [23]. In agreement with a study from the FMOH report [15], this study indicated that there was a significant difference in the labeling of medicine practice between the two facilities.

Against our expectations, findings revealed that 90.2% of Non-APTS respondents were able to recall the right dosage time of the medicines but only 2.1% of medicines were adequately labeled. The first possible reason is that the evaluation of knowledge was done immediately after the dispensing process. Therefore the influence of adequate labeling on patient medicine knowledge seems unimportant. Another possible explanation is that 93% of dispensed prescriptions at Non-APTS hospitals were labeled with only the dosage time of the medicines. According to WHO, the medicine packages label must include all the three pieces of information (patient name, drug name, and frequency of administrations) [9]. The result was lower than percentage of medicines adequately labeled in Kuwait (66.9%) [26], Swaziland (55.9%) [36], Pakistan (100%) [22], China (95%) [31]. However, in both hospitals the percentage of medicines sufficiently labeled was too much lower than the ideal WHO standard value (100%). This could be explained by reasons like lack of packaging materials, high patient load and lack of knowledge in the labeling of dispensed medicines.

### Prescribing indicators

WHO highly recommends the mean number of medicines per prescription in a variation from 1.6 to 1.8 as safety precautions for patients because it minimizes the risk of adverse drug reactions, decrease drug-drug interactions and decrease out of pocket costs for patients. The mean number of medicines per prescription in APTS and non-APTS facilities was 2.3 and 2.8 drugs per encounter, respectively. There was significant variation between the two health care facilities. However, this finding is higher than the acceptable ideal WHO range of 1.6–1.8 drugs per encounter. This high number of drugs per prescription might be due to lack of sufficient knowledge of prescribers, unavailability of standard treatment guidelines, shortage of therapeutically right drugs and financial incentives to the prescribers. The result also shows that a higher number of drugs per prescription when compared to other reported studies in Malawi (1.8) [25], Zimbabwe (1.3) [27]. However, figures that are higher than these results were documented in different areas such as 4.8 in Ghana [39], 5.2 in Nigeria [36], 3.5 in India [40], and 2.9 in southern Brazil [41].

The percentage of encounter with antibiotics prescribed was 57.8% in APTS facilities and 56% in Non-APTS facilities which is well high compared to the ideal WHO standard (20.0–26.8). However, the variation between the two health care facilities was statistically insignificant.This finding was higher than percentage of encounter with antibiotics prescribed in Malawi (34%) [25], Saudi Arabia (23%) [21], Bangladesh (24.7) [32], Pakistan (48.9%) [22] and Bahrain (28%) [42].

The high value in this study might be due to a lack of in-service training for prescriber or dispenser, the patient expectation to receive antibiotics or health care providers’ belief that low efficacy of antibiotics. Irrational use of antibiotics can cause unwanted drug reactions, hospital admission [43] and antibiotic-resistant strains of bacteria [44]. The high percentage of encounters with antibiotics prescribed in APTS hospitals indicated that the program needs to be improved by incorporating additional package of interventions that address rational prescribing practices.

The findings of this study illustrated that the percentage of prescriptions with an injection encounter was found to be 38.2% in the primary hospitals with APTS and 40.5% in Non-APTS primary hospitals. The difference between the two hospitals was not statistically significant. The percentage of encounter with injection was found to be excessively high in both hospitals compared to WHO a criterion which is varied from 13.4 to 24.1 [9]. The potential reason for the too high prescribing practice of injections could come from attitudes and beliefs of prescribers and patients about the efficacy of injection in comparison with other routes of administrations. In fact, injections are crucial dosage forms in certain emergency situations because of their onset of action. However, excessive use of injections may be related to the risk of blood-borne diseases like hepatitis and HIV/AIDS [45], physiological and psychological pain [9] during administration. Study findings from north Ethiopia revealed that knowledge regarding the safe practices of injection administration was suboptimal among injection prescribers and providers [46]. Therefore, lack of safe injection practices and indiscriminate uses of injections (nonadherence to universal precautions) can increase the risk of spreading blood-borne diseases such as HIV/AIDS and hepatitis. In addition,injections are always more expensive than equivalent oral dosage forms [47]. Our value is higher than reported in Burundi (10%) [48] and Botswana (9%) [49]. However, in some countries, this percentage was even higher than our results such as 80% in Ghana [39] and 41.8% in Cameroon [50]. This difference could be enlightened by variations in morbidity and level of care. This finding suggests that the APTS program is not associated with the rational injection prescribing practice. WHO highly advocates prescribing medication by using the generic of the medication.It gives clear identification of the drug, decrease out of pocket expenditure for medicine and promote effective communication among health professionals [9]. The percentage of medicines prescribed by the nonproperty name was found to be 90% in APTS and 91% in non-APTS. The variation was statistically significant. Our result was lower than the ideal WHO standard (100%) [9].Our value is also lower than reported in primary health facilities in eastern Ethiopia (97%) [30].

### Complementary indicators

The current study revealed that outpatient pharmacy users in APTS facilities reported higher perceived quality of pharmacy service for almost all satisfaction items except for respect shown by the dispenser, fairness of cost of medicines, dispensers’ professionalism and information on the storage of medicines when compared with those in non-APTS facilities. There was also a significantly increased total satisfaction score among patients served at APTS facilities compared to non-APTS. These findings were analogous to a study conducted at the national level which found that patients were more satisfied in all aspects of pharmacy service in APTS hospitals than Non-APTS hospitals except fairness of cost [15]. This variation was there when the different patient characteristics were adjusted thus shows an important effect of the program in providing good pharmacy service. The five elements of APTS may contribute to the increased total satisfaction score, including, bin ownership, effective workforce deployment, transparent transaction, pharmacy workflow reorganization and dispensing setup rearrangement. More than half of the participants were very dissatisfied with the availability of prescribed drugs and waiting time in Non-APTA facilities. This is attributed to the poor inventory management and disorganized workflow in the pharmacy. In Non-APTS hospitals, the dispensary has one grilled window through which patients were served. Only one or two pharmacists were giving service through a window without the privacy of patients to receive counseling and ask questions. Moreover, a patient must queue at least three times; first—to get the prescription evaluated, price of medicines confirmed in one of the grilled window; second- to pay the price at the finance window (usually outside the pharmacy); and third to collect the prescribed medicines and to get counseling by coming back to the dispensary grilled window. To do so, patients were observed standing with a long queue waiting for services before they see a pharmacist or a cashier or in an environment with inadequate patient waiting areas, shelter and chairs to sit.

In line with evidence from Gonder, north Ethiopia [51], this study indicated that almost 40% of patients in both facilities very dissatisfied with information on the storage of medicines. This study also found that most patients from both APTS and Non-APTS primary facilities were dissatisfied with the fairness of the cost of medicine. This finding was similar to a study conducted by FMOH which found that most patients were dissatisfied with the cost of medicines in both sites [15].

The result of this study also revealed that the average cost of the medicine per encounter in the primary hospitals with the APTS (41.35 Ethiopian Birr/1.48 United States Dollar) was lower than at the hospitals without APTS (45.63 Ethiopian Birr/1.63 United States Dollar). At the time of the study, the lowest paid unskilled governmental worker earned 1200 Ethiopian Birr per month (42.86 United States Dollar/month) as at 2017. The cost of drugs is considered affordable if it quantity to the salary of one workday or less of the lowest paid unskilled government worker, for one course of treatment [52]. On average, the lowest unskilled governmental worker needed a 1.14 and 1.03 day’s wages to cover the drug cost in non-APTS and APTS facilities, respectively. In both facilities, the lowest unskilled governmental worker costs for medicine higher than their daily wage. Although the lowest paid unskilled governmental worker wage was considered as a measure of affordability it is likely that a considerable part of the Ethiopian population gets less. The high cost of medicines per encounter at both APTS and Non-APTS primary hospitals might be due to the high number of drugs prescribed per prescription, excessive prescribing by nonproperty name, minimal prescribing from the essential medicine list and low salary of the lowest unskilled governmental worker. This implies that APTS interventions are not effective in reducing the average cost of the drug per encounter. Our finding is similar to the findings from the study done on the affordability and prices of medicines in Guatemala [53] and Malawi [54].

### The implication of the study

This research has added to the evidence that the APTS program related with better WHO patient care indicators(dispensing time, availability of prescribed medicines and knowledge about medicines), health facility indicators (availability of essential medicines and availability of a copy of EDL) and complementary indicators (patient satisfaction toward the pharmacy services). This implies that the APTS program is appropriate to improve patient care indicators, health facility indicators, and patient satisfaction. Therefore APTS needs to be adopted as the standard dispensing practice in all Ethiopian primary hospitals. The pharmacy colleges must take the responsibility to educate their trainees on the way to practice APTS.

To achieve significant improvement on medicine use indicators such as percentage of medicines adequately labeled, number of medicines per encounter, percentage of medicines ordered by nonproperty name, percentage of encounter with antibiotics and percentage of encounters with an injections at APTS primary hospitals, this research strongly suggest that the government health sector develop health policies aimed at establishing Electronic Health Information System (EHIS) that embedded with Clinical Decision Support System (CDSS). There is also a need to strengthen the system for continuing professional development of prescribers to ensure that they acquire the required knowledge and skills to prescribe rationally. Our results also point to the need for policymakers to focus their concentration on strategies that address strengthing interprofessional education (STRIPE) to promote communication and negotiation skills, understanding of the professionals’ roles, and enhance patient-centered care.

### Study strengths and limitations

The strength of this research is, it used validated WHO instruments to compare drug use between APTS and non-APTS primary health care facilities. It is the first study comparing WHO drug use indicators among primary hospitals with and without the APTS program.

Despite its strengths, there are some limitations to this study. Because of the nature of the comparative cross-sectional study design; causal relationships cannot be made. Control health care facilities were, however, matched by services and administrative type.

The results of this study only compare patient care and prescribing indicators in the outpatient department of primary health care facilities. Our study does not reveal the medicine used in inpatient wards of the healthcare facilities. Performing interviews in the location near the dispensing room or within the health care facility might have motivated patients to give more positive responses than their real experience. We did not control for clustering at dispenser type (pharmacist or pharmacy technician). Since primary hospitals varied according to catchment size it is possible other differing aspects, such as financing, could influence drug use indicators. The findings of this study were not supported by the qualitative study design.

## Conclusions

This study revealed that patient care indicator values, the level of patient satisfaction on the pharmacy services and health facility indicator values were significantly better in APTS primary level hospital than Non-APTS primary level hospitals.This implies that APTS is appropriate and needs to be adopted as a standard dispensing practice in all primary level hospitals. However, most of WHO stated prescribing indicators and labeling practices were not met by both APTS and Non-APTS facilities included in the study. This indicates that the APTS program should include additional tools, methods and strategies (managerial, educational and regulatory) to reverse the existing irrational prescribing and inadequate labeling practices and improve the drug use pattern on these primary facilities and the country’s health care system in general.

## Supporting information

S1 File Data collection tool. (DOCX)

## Abbreviations

APTS: Auditable Pharmaceutical Transactions and Services
CDSS: Clinical Decision Support System
CI: Confidence Interval
DTC: Drug and Therapeutics Committee
DTP: Drug and Therapeutic Problem
EDL: Essential Drug List
EHIS: Electronic Health Information System
ETB: Ethiopian Birr
FMOH: Federal Ministry of Health
HSDP: Health Sector Development Program
INRUD: International Network of Rational Use of Drugs
Non-APTS: Non Auditable Pharmaceutical Transactions and Services
OPD: Outpatient Department
SD: Standard Deviation
SIAPS: Systems for Improved Access to Pharmaceuticals and Services
SNNPR: Southern Nation Nationality and People Republic
SPSS: Statistical Packages for Social Sciences
STRIPE: Strengthening Interprofessional Education
USAID: United States Agency for International Development
WHO: World Health Organization
