# Supplementary material for: A cross sectional comparison of drug use indicators using WHO methodology in primary level hospitals participating in an Auditable Pharmaceutical Transactions and Services program versus non-APTS primary hospitals in Southern Ethiopia
Source: PLoS One. 2019 Oct 7;14(10):e0223523. doi: 10.1371/journal.pone.0223523 (PMC6779258; doi:10.1371/journal.pone.0223523)
Supplement: S1 File — (DOCX) [file pone.0223523.s001.docx]

**English version of data collection tools**

## English version information sheet and consent form

## Information sheet

Name of Investigators: Biruk Wogayehu and Yilima Chisha

Name of organization: Arbaminch College of Health Sciences

Introduction: This information sheet & consent form is prepared by the principal investigators whose aim is to compare of drug use indicators using WHO methodology in primary level hospitals participating in an Auditable Pharmaceutical Transactions and Services program versus non-APTS primary hospitals in Southern Ethiopia.Others that will be participated in this research include five trained clinical pharmacists for supervision, and twenty six pharmacy technician for data collection.

**Purpose of the research project**: Even though evidence suggests that APTS program has the capacity for improving pharmaceutical transactions and services at the secondary and tertiary level health care facilities, has not particularly study whether APTS program affect the medicine prescribing practices, dispensing time, availability of prescribed medications and affordability of prescribed medicines, the most crucial elements in the rational drug use practice. Moreover, in Ethiopia, there have been no studies that analyzed the APTS program at primary level health care facility. This study tries to fill that limitation by comparing Ethiopia APTS program primary health care facilities with an analogous cross-section of primary level health facilities without APTS.

Procedure: This study will be conducted mainly on patients who had their medicines orders filled at outpateint pharmacy of the hospital were included in the study. Before starting the research, ethical approval for the research was obtained from the Institutional Ethics Review Board (IRB) of Arbaminch College of Health Sciences. Authorized permission letter was obtained from selected areas health offices and the data collection started after cooperation letter was written to all twenty primary level hospitals. Patient care indicators and satisfaction will be evaluated prospectively by interviewing and observing patients from each group. Health care facilities will be evaluated through observation.

Risk and or discomfort: by participating in this research project you will not feel any discomfort and the interview will last only 20 to 30 minutes. Information gathered will be kept confidently. There is no risk at all by participating in this research.

Benefits: your participation in this research project, may not give you a direct benefit. But it has great role to achieve improvements in the pharmacy service delivery.

Incentives: you will not get paid or have any other incentives to take part in this project.

**Confidentiality and anonymity:** The information we collate form this research will be kept confidential. Information about you that will be collected from this study will be stored in a file, which will not have your name on it. The investigator will store by locking so that unintended body will not have accesses to it. Your name will not be written on this form & used in connection with any of the information you tell me.

Right to refuse and/ withdraw: you have full right to refuse form participating in this research you can choose not to respond some or all of the questions. This will not affect your health service delivery that you got form the hospital.

## Consent form:

Hello! My name is ______________________I am working in the research team .I would like to gather information regarding the level of patients’ health care satisfaction with pharmacy service delivery system and knowledge about prescribed medicine while you are in this health institution which is very important to improve the service delivery. Your cooperation and willingness to give the information is helpful in identifying problems related to the concern. We have identified you as a study participant believing that you would be willing to respond to questions to give me your sincere and truthful answers. By participating in this research project, your name or address will never be written in this form. All information that you give will be kept strictly confidential. Your participation is voluntary and you are not obliged to answer any question you do not wish to answer. If you have any question or anything unclear, please feel free to ask. If you are not still comfortable along the discussion process you have full right to drop it any time you want. You will not be asked to give reasons and you will not miss any chance of service delivery from the hospital for doing so.

If you are clear with the information provided, do I have your permission to continue?

1. If yes_______________ thank you, continue to the next page.
2. If no________________ thank you, stop.

#

# Structured English Version Questionnaire

## Arbaminch College of Health Sciences

Questionnaire developed for data collection of patients’ satisfaction on pharmacy service delivery at______________primary level hospital

### Identification:

Name of the primary hospital_______________________ Code No.___________

Date of interview________________ time started ________time finished_______

Interviewer or supervisor who certified that, informed consent has been given from the respondents.

Name of interviewer _________________________ Signature ____________

Name of supervisor __________________________ signature _____________

**Part-1 Socio-demographic and health-related characteristics**

*I will ask you some questions to gather information about your socio-demographicand health-related characteristics*

**Reapondent code:** _______________________

| **S.No.** | | **Question** | **Response** |
| --- | --- | --- | --- |
| 101 | | How old are you? | ____________Years |
| 102 | | Sex | - Male - Female |
| 103 | | Where is your residence address? | - Urban - Rural |
| 104 | | What is your religion? | - Orthodox - Muslim - Protestant |
| 105 | | What is your marital status? | - Single - Married - Divorced - Windowed |
| 106 | | What is your occupation? | - Government employee - Private employee - Farmer - Merchant - No job |
| 107 | | What is your educational level? | - Not able to read and write - Primary school - Secondary school - Higher education |
| 108 | | What is your payment status? | - Free - Cash/credit |
| 109 | | What is your health status? | - Good - Bad |
| 110 | | Service sought for | - Self - Other |
| 111 | Number of visist | | - First vist - Second visist - Follow up |

**Part-2 Instrument to evaluate patients’ level of knowledge about drug prescription.**

The following questions refer to medications that you will take or are ready taking.Do not worry about answering them correctly, because all answers are welcome.

If there is more than one prescribed medications, the first one in the medical prescription received will be considered for the following questions to be answered.

| **S.No.** | **Question** | **Response** |
| --- | --- | --- |
| 201 | What is the name of prescribed medication? | 1. Does not know 2.______ (response) |
| 202 | What is the dosage of medication that you should take? | 1. Does not know 2.______ (response) |
| 203 | What are the times that you take the medication? | 1. Does not know 2.______ (response) |
| 204 | For how long should youtake the prescribed medication? | 1. Does not know 2.______ (response) |
| 205 | How should you use the prescribed medication? | 1. Does not know 2.______ (response) |
| 206 | What should you do if you miss one or more dosage? | 1. Does not know 2.______ (response) |
| 207 | Is there another medication, food or beverage that you should avoid while using this medication? | 1.If yes,which?______(response) 2.No  3.Does not know |
| 208 | Can this medication cause side effects? | 1.If yes,which?______(response) 2.No  3.Does not know |
| 209 | Where should you store the prescrbed medication? | 1. Does not know 2.______ (response) |

**Part-3** **Patient satisfaction with the pharmacy services they received**

*I will ask you some questions to gather information about your satisfaction toward pharmacy service*

| **Serial No.** | **Question** | **Response** |
| --- | --- | --- |
| 301 | Did you feel that the location of pharmac reference to other services was convenient for you? | 1. Strongly disagree 2. Disagree 3. Neutral 4. Agree 5. Strongly agree |
| 302 | You get adequate provision of information for location of pharmacy | 1. Strongly disagree 2. Disagree 3. Neutral 4. Agree 5. Strongly agree |
| 303 | You feel satisfied with cleanliness of outpatient pharmacy | 1. Strongly disagree 2. Disagree 3. Neutral 4. Agree 5. Strongly agree |
| 304 | You feel satisfied with the space of the dispensary | 1. Strongly disagree 2. Disagree 3. Neutral 4. Agree 5. Strongly agree |
| 305 | You feel satisfied with the time spent waiting to be seen by dispenser? | 1. Strongly disagree 2. Disagree 3. Neutral 4. Agree 5. Strongly agree |
| 306 | You are satisfied with information on how to take your medication to you? | 1. Strongly disagree 2. Disagree 3. Neutral 4. Agree 5. Strongly agree |
| 307 | You are satisfied with clarification of all possible side effects or adverse events to you? | 1. Strongly disagree 2. Disagree 3. Neutral 4. Agree 5. Strongly agree |
| 308 | You are satisfied with information on contraindications to you? | 1. Strongly disagree 2. Disagree 3. Neutral 4. Agree 5. Strongly agree |
| 309 | You are satisfied with information on storage of medicines to you? | 1. Strongly disagree 2. Disagree 3. Neutral 4. Agree 5. Strongly agree |
| 310 | You are satisfied with labeling information on dispensed drugs to you? | 1. Strongly disagree 2. Disagree 3. Neutral 4. Agree 5. Strongly agree |
| 311 | Do you agree that you get all the prescribed drugs oredered to you? | 1. Strongly disagree 2. Disagree 3. Neutral 4. Agree 5. Strongly agree |
| 312 | You feel satisfied with the fairness of cost of drugs in the outpatient pharmacy | 1. Strongly disagree 2. Disagree 3. Neutral 4. Agree 5. Strongly agree |
| 313 | Your feelings of the quality of drug dispensed to you? | 1. Strongly disagree 2. Disagree 3. Neutral 4. Agree 5. Strongly agree |
| 314 | You are served with respect of the dispenser? | 1. Strongly disagree 2. Disagree 3. Neutral 4. Agree 5. Strongly agree |
| 315 | The necessary measures were taken to  Assure your privacy during examinations? For  example, a private room, screened area, etc | 1. Strongly disagree 2. Disagree 3. Neutral 4. Agree 5. Strongly agree |
| 316 | During your communication with the dispenser ,professional relationship | 1. Strongly disagree 2. Disagree 3. Neutral 4. Agree 5. Strongly agree |
| 317 | Dispenser is willing to answer your question? | 1. Strongly disagree 2. Disagree 3. Neutral 4. Agree 5. Strongly agree |
| 318 | You feel satisfied with the amount of time the dispenser spends with you | 1. Strongly disagree 2. Disagree 3. Neutral 4. Agree 5. Strongly agree |

**Part-4 Prescribing indicator form and complementary indicator (Cost)**

**Location: ______________________________________________________**

**Investigator: _____________________________________________________ Date: ______**

***0=No and 1=Yes**

| **Seq.**  **No.** | **Code** | **Date of**  **prescribed** | **Number of drugs**  **prescribed** | **Number of drugs with generic name** | **Antibiotics**  **(0/1)*** | **Injection**  **(0/1)*** | **Number of drugs from EDL** | **Cost** |
| --- | --- | --- | --- | --- | --- | --- | --- | --- |
|  |  |  |  |  |  |  |  |  |
|  |  |  |  |  |  |  |  |  |
|  |  |  |  |  |  |  |  |  |
|  |  |  |  |  |  |  |  |  |
|  |  |  |  |  |  |  |  |  |
|  |  |  |  |  |  |  |  |  |
|  |  |  |  |  |  |  |  |  |
|  |  |  |  |  |  |  |  |  |
|  |  |  |  |  |  |  |  |  |
|  |  |  |  |  |  |  |  |  |
|  |  |  |  |  |  |  |  |  |
|  |  |  |  |  |  |  |  |  |
|  |  |  |  |  |  |  |  |  |
|  |  |  |  |  |  |  |  |  |
|  |  |  |  |  |  |  |  |  |
|  |  |  |  |  |  |  |  |  |
|  |  |  |  |  |  |  |  |  |
|  |  |  |  |  |  |  |  |  |
|  |  |  |  |  |  |  |  |  |
|  |  |  |  |  |  |  |  |  |
|  |  |  |  |  |  |  |  |  |
|  |  |  |  |  |  |  |  |  |
|  |  |  |  |  |  |  |  |  |
|  |  |  |  |  |  |  |  |  |
|  |  |  |  |  |  |  |  |  |
|  |  |  |  |  |  |  |  |  |
|  |  |  |  |  |  |  |  |  |
|  |  |  |  |  |  |  |  |  |
|  |  |  |  |  |  |  |  |  |
|  |  |  |  |  |  |  |  |  |
|  |  |  |  |  |  |  |  |  |
| **Total** |  |  |  |  |  |  |  |  |

**Part-5 Patient care form**

**Location: ______________________________________________________**

**Investigator: _____________________________________________________ Date: ______**

| **Seq.No.** | **Patient code** | **Consulting time(minutes)** | **Dispensing time(secs)** | **Number of drugs dispensed** | **Number of drugs adequately labeled** |
| --- | --- | --- | --- | --- | --- |
|  |  |  |  |  |  |
|  |  |  |  |  |  |
|  |  |  |  |  |  |
|  |  |  |  |  |  |
|  |  |  |  |  |  |
|  |  |  |  |  |  |
|  |  |  |  |  |  |
|  |  |  |  |  |  |
|  |  |  |  |  |  |
|  |  |  |  |  |  |
|  |  |  |  |  |  |
|  |  |  |  |  |  |
|  |  |  |  |  |  |
|  |  |  |  |  |  |
|  |  |  |  |  |  |
|  |  |  |  |  |  |
|  |  |  |  |  |  |
|  |  |  |  |  |  |
|  |  |  |  |  |  |
|  |  |  |  |  |  |
|  |  |  |  |  |  |
|  |  |  |  |  |  |
|  |  |  |  |  |  |
|  |  |  |  |  |  |
|  |  |  |  |  |  |
|  |  |  |  |  |  |
|  |  |  |  |  |  |
|  |  |  |  |  |  |
|  |  |  |  |  |  |
|  |  |  |  |  |  |
|  |  |  |  |  |  |
|  |  |  |  |  |  |
|  |  |  |  |  |  |
|  |  |  |  |  |  |
|  |  |  |  |  |  |

**Part-6 Health facility indicator form (OBSERVATION CHECKLIST)**

Facility name: ____________________________ Date______________

Name of data collector_______________________________ Time of data collection: _____ hr.: min

**Section A. Availability of the key medicines in the dispensaries during the day of visit for facility APTS-1(Different key medicines for each facility based on their top ten diseases)**

| **No.** | **Medicine name** | **Availability in store** | |
| --- | --- | --- | --- |
|  |  | **Yes=1** | **No=0** |
|  | Amoxicillin 125mg/5ml syrup/suspension |  |  |
|  | Amoxicillin 250mg/500mg cap/tab |  |  |
|  | Ceftriaxone 500mg/ 1g inj |  |  |
|  | Ciprofloxacin 500mg caps/tab |  |  |
|  | Sulphamethoxazole + Trimethoprim 200mg + 40mg in 5ml |  |  |
|  | Arthmeter + Lunfanthrine |  |  |
|  | Mebendazole oral suspension,100mg/5ml |  |  |
|  | Metronidazole 250mg cap/tab |  |  |
|  | Atenolol 50mg tab |  |  |
|  | Enalapril 5/10mg tab |  |  |
|  | Hydrochlorothiazide 25mg tab |  |  |
|  | Metformin 500mg tab |  |  |
|  | Simvastatin 20mg tab |  |  |
|  | Diazepam 5mg tab |  |  |
|  | Amitriptyline 25mg tab |  |  |
|  | Fluoxetine 20mg cap |  |  |
|  | Phenobarbitone 100mg tab |  |  |
|  | Haloperidol tab |  |  |
|  | Omeprazole 20mg cap |  |  |
|  | Salbutamol inhalers |  |  |
|  | Oral rehydration salts (ORS)/zinc |  |  |
|  | Diclofenac sodium 50mg tab |  |  |
|  | Paracetamol 120mg/5ml |  |  |
|  | Sodium chloride 0.9% (normal saline) |  |  |
|  | Oxytocin 10 IU |  |  |
|  | Magnesium sulphate inj. |  |  |
|  | Ferrous sulphate + folic acid tab |  |  |
|  | Oral contraceptives tab |  |  |
|  | Vitamin K 10 IU |  |  |
|  | Tetracycline eye ointment |  |  |

**Section B. Day outstock of the key medicines in the dispensaries**

| **No.** | **Medicine name** | **Number of day**  **out of stock** |
| --- | --- | --- |
|  |  |  |
|  | Amoxicillin 125mg/5ml syrup/suspension |  |
|  | Amoxicillin 250mg/500mg cap/tab |  |
|  | Ceftriaxone 500mg/ 1g inj |  |
|  | Ciprofloxacin 500mg caps/tab |  |
|  | Sulphamethoxazole + Trimethoprim 200mg + 40mg in 5ml |  |
|  | Arthmeter + Lunfanthrine |  |
|  | Mebendazole oral suspension,100mg/5ml |  |
|  | Metronidazole 250mg cap/tab |  |
|  | Atenolol 50mg tab |  |
|  | Enalapril 5/10mg tab |  |
|  | Hydrochlorothiazide 25mg tab |  |
|  | Metformin 500mg tab |  |
|  | Simvastatin 20mg tab |  |
|  | Diazepam 5mg tab |  |
|  | Amitriptyline 25mg tab |  |
|  | Fluoxetine 20mg cap |  |
|  | Phenobarbitone 100mg tab |  |
|  | Haloperidol tab |  |
|  | Omeprazole 20mg cap |  |
|  | Salbutamol inhalers |  |
|  | Oral rehydration salts (ORS)/zinc |  |
|  | Diclofenac sodium 50mg tab |  |
|  | Paracetamol 120mg/5ml |  |
|  | Sodium chloride 0.9% (normal saline) |  |
|  | Oxytocin 10 IU |  |
|  | Magnesium sulphate inj. |  |
|  | Ferrous sulphate + folic acid tab |  |
|  | Oral contraceptives tab |  |
|  | Vitamin K 10 IU |  |
|  | Tetracycline eye ointment |  |

**Amharic version of data collection tools**

Amharic version information sheet and consent form

**የተመራማሪዉ ስም፡** ብሩክ ወጋየሁ እና ይልማ ቺሻ

**የድርጅቱ ስም፡** አርባምንጭ ጤና ሳይንስ ኮሌጅ

**መግቢያ፡**ይህ ስምምንነት የተዘጋጀዉ በዋናዉ ተመራማሪ ነዉ፡፡የዚህ ጥናት አላማ የመዳኒት አጠቃቀምን ኤፕትኤስን በጀመሩ ተቆማትና ባልጀመሩ በደቡብ ዉስጥ ያሉ ተቆማት መካከል ያለዉን ንጽጽር ለማየት ነዉ፡፡በተጨማሪም ሁለት የጤና መኮንኖች እንደ ት ስድስት የፋርማሲ ባለሞያዎች በመረጃ ስብሳቢነት ይሳተፋሉ፡፡

**የጥናቱ አላማ**፡ከዚህ ቀደም ኤፕትኤስ የመዳኒት አጠቃቀምን እደሚያሻሽል በአጠቃልይና እሪፈራል ሆስፒታሎች ላይ የተደረገዉ ጥናት ያሳያል፡፡ነገር ግን ይህ ፕሮግራም በመዳኒት አስተዛዝ ላይ፤በምክር አገልግሎት ስዓት ላይ፤የታዘዙ መዳኒቶች ስለመገየታቸዉ እና የመዳኒት ዋጋ ተመጣጣይነት ላይ ያመጣቸዉ ለዉጦች እስካዉን አልተዳሰሱም፡፡

**ፕሮሲጀር፡** ይህ ጥናት በዋነያነት ትኩርት የሚያደርገዉ በአዉት ፔሸንት ፋርማሲ ተጠቃሚ በሽተዮች ላይ ነዉ፡፡ይህን ጥናት ለማከናወን ከአርባምንጭ ጤና ሳይንስ ኮሌጅ ፍቃድ ተቶታል፡፡ለዚህ ጥናት መረጃ የሚሰበሰበዉ ከመዳኒት ማዘዣ ወረቀት እና ከበሽተዮች ጋር በሚደረግ ቃለ ምልልስ ነዉ፡፡

**አደጋ ወይም ስጋት፡**በዚህ ጥናት ላይ በመሳተፎ ምንም አይነ**ት** ጉዳት አይደርስቦትም፡፡የአጥናቱ ቃለ ምልልስ ከ 20 እስከ 30 ደቂቃ ቢፈጅ ነዉ፡፡

**ጥቀም፡**በዚህ ጥናት ላይ በመሳተፎ የሚያገዩት ግላዊ የሆለ ጥቅም የለም፡፡የጥናቱ ዉጤት የፋርማሲን ሙያ ለማሻሻል ግን ትልቅ ሚና አለዉ፡፡

**ክፍያ፡**በዚህ ጥናት ላይ በመሳተፎ የሚያገዩት ልዩ ክፍያ የለም፡፡

**የመረጃ ሚስጥራዊነት፡** ለዚህ ጥናት የሚሰበሰቡ መረጃዎች ከበሽተያዉ ፍቀድ ከተገየ ነዉ፡የተሰበሰቡ መረጃዎች ጥብቅ በሆነ ቦታ ነዉ የሚቀመጡት፡፡

**ያለመሳተፍ መብት፡**በጥናቱ ላይ ያለመሳተፍ መብት አላቸዉ፡፡በዚህ ጥናት ላይ ባለመሳተፎ በሚያገዩት አገልግሎት ላይ ምንም አይነት ተጽኖ አያሳድርም፡፡

**ኮንሰንት ፎርም**

እ”ÅU” ›Å\/ªL‹G<;

እኔ eT@---------------- ¾}vMŸ< u›`vU”ß Ö?“ dÃ”e ¢K?Ï uT>Å[Ñ¬ ¾Ø“ƒ“ U`U` ›vM eJ” ›G<” MÖÃqƒ ¾UðMÑ¬ uó`Tc= ¡õM ÁÑ¿ƒ ›ÑMÓKAƒ LÃ ÁKAƒ” ›`ካታና ስታዘዘሎት መዳኒት ያሎት እዉቀት በተመለከተ ነዉ፡፡የእርሶ ትብብርና ፍቃደይነት ለጥናቱ መሳካት ትልቅ ፋይዳ አለዉ፡፡በዚህ ጥናት ላይ በሚሳተፉበት ወቅት ማንነቶን የሚገለጽ መረጃ አይሰበሰብም፡፡በጥናቱ ላይ እዲሳተፉ የሚያስገድዶት ሰዉ የለም፡፡የሚሰጡት መረጃ ሚስጠራዊነቱ የተጠበቀ ነዉ፡፡በመሃል ያልገባዎት ወይም ግልጽ ያልሆነ ነገር ካለ በለጻነተ መጠየቅ የቻላል፡፡በመሃል ምቾት ካል ተሰማዎት በማነያዉም ስዓት የማቆረጥ መብት አሎት፡፡ለምን እዳቆረጡ የሚጠይቆት ሰዉ የለም በተጨማሪም በማቆረጦ የሚያጡት ምንም ዓይነት የህክምና አገልግሎት የለም፡፡

ከላይ የቀረበዉ መረጃ ግልጽ ከሆነ፤ፍቃደያ ከሆኑ መቀጠል እችላለዉ?

1. ከተስማሙ--------------አመስግናለዉ፤ወደ ሚቀጥለዉ መሄድ እንእላለን
2. ካል ተስማማሙ----------አመሰግናለዉ፣እዚ ላይ አበቃለዉ፡፡

# Structured Amharic Version Questionnaire

አርባምንጭ ጤና ሳይንስ ኮሌጅ

ይህ የመጠይቃ ቅጽ በሽተዪች በ----------------ሆስፒታል የፋርማሲ አገልግሎት ላይ ያላቸዉን እርካታና ስለታዘዘላቸዉ መዳኒት ያላቸዉን እዉቀት ለመለካት ነዉ፡፡

የሆስፒታሉ ስም--------------------- ሚስጥራዊ ቁጥር-------------

ቀን--------------------- የተጀመረበት------------ የተጠናቀቀበት------------

የመረጃ ስብሳቢዉ ስም--------------------------- ፊርማ------------------------

ሱፐርቫይዘር ስም--------------------------------- ፊርማ------------------------

ከፍል-1 TIu^© Ñ<ÇÄ‹”“ Ö?“” u}SKŸ}

| }.l | ØÁo | ULi |
| --- | --- | --- |
| 101 | እድሜሆ ስንት ነዉ | ---------------¯Sƒ |
| 102 | ïታ | - ¨”É - c?ƒ |
| 103 | ¾T>•\ƒ ¾ƒ ’¬ | - Ÿ}T - ÑÖ` |
| 104 | HÃT•„ | - *`„Ê¡e - S<eK=T> - ýaስታንት |
| 105 | ¾Òw‰ G<’@ታ  | - ÁLÑv/‹ - ÁÑv/‹ - ¾}óታ/ች - ¾V}uƒ/vƒ |
| 106 | ¾Y^ G<’@ታ | - ¾S”Óeƒ }k×] - ¾ÓM }k×] - Ñu_ - ’ÒÈ - Y^ ›Ø |
| 107 | ¾ƒUI`ƒ Å[Í | - T”uw“ Séõ ¾TÃ‹M/¾Tƒ‹M - ¾SËS]Á Å[Í - G<K}Á Å[Í - Ÿõ}Á ƒUI`ƒ |
| 108 | ¾¡õÁ G<’@ታ | - ’é - u¡õÁ/uwÉ` |
| 109 | ¾Ö?“ G<’@ታ ሁኔታሆ ምን ይመስላላል | - Å“ ’Ã - SØö G<’@ታ |
| 110 | አገልግሎት ያገየሁት | - K^c? - KK?L c¬ |
| 111 | ለስንተያ ግዜ ነዉ የመጡት- | - KSË]Á - KG<K}Á - }SLLi |

ክፍል-2 በሽተዮች ስለታዘዘላቸዉ መዳኒት ያላቸዉን እዉቀት ለመለካት የተዘጋጀ መጠይቅ ነዉ፡፡

| **ተ.ቁ** | **ጥያቄ** | **ምላሽ** |
| --- | --- | --- |
| 201 | የታዘዘሎት መዳኒት ስም | 1.አላዉቅም  2.----------(ምላሽ) |
| 202 | ምን ያህል ነዉ በአንዴ የሚወስዱት | 1.አላዉቅም  2.----------(ምላሽ) |
| 203 | መቼ መቼ ነዉ የታዘዘሎትን መዳኒት የሚወስዱት | 1.አላዉቅም  2.----------(ምላሽ) |
| 204 | ለምን የህል ግዜ ነዉ የታዘዘሎትን መዳኒት የሚወስዱት | 1.አላዉቅም  2.----------(ምላሽ) |
| 205 | እንዴት ነዉ የታዘዘሎትን መዳኒት የሚወስዱት | 1.አላዉቅም  2.----------(ምላሽ) |
| 206 | በአጋጣሚ መዉሰድ ባለቦት ስዓት መዳሊቶን ባይወስዱ ምን ያደርጋሉ | 1.አላዉቅም  2.----------(ምላሽ) |
| 207 | አሁን ከታዘዘሎት መዳኒት ጋር መወሰድ የሌለበት መዳኒ፤ምግብ ወይም መጠጥ አለ | 1.አዎ ካሉ፤ትዮቹ---------  2.የለም  3.አላዉቅም |
| 208. | የታዘዘሎት መዳኒት  የጎንዮሽ ጉዳት አለዉ | 1.አዎ ካሉ፤ትዮቹ---------  2.የለም  3.አላዉቅም |
| 209 | የታዘዘሎት መዳኒት  በየት ቦታ ያስቀምጡታል | 1.አላዉቅም  2.----------(ምላሽ) |

**ክፍል-3 በፍርማሲ አገልልግሎት ላይ በሽተዮች ያላቸዉን እርካታ ለመመለካት የተዘጋጀ መጠይቅ**

| **ተ.ቁ** | **ጥያቄ** | **መልስ** |
| --- | --- | --- |
| 301 | ፍርማሲ የሚገይበት ቦታ ከሌሎች አገልግሎቶች በቅርብ እርቀት ላይ ይገያል | 1. በጣም አልስማማም 2. አልስማማም 3. ምንም አልልም 4. እስማማላዉ 5. በጣም እስማማለዉ |
| 302 | ፍርማሲ የሚገይበት ቦታ የሚጦቁም መረጃ አግይቻለዉ | 1. በጣም አልስማማም 2. አልስማማም 3. ምንም አልልም 4. እስማማላዉ 5. በጣም እስማማለዉ |
| 303 | በፍርማሲ ክፍል ንጽህና እረክቻለዉ | 1. በጣም አልስማማም 2. አልስማማም 3. ምንም አልልም 4. እስማማላዉ 5. በጣም እስማማለዉ |
| 304 | የፍርሲ ክፍል ስፋቱ ምቹ ነዉ | 1. በጣም አልስማማም 2. አልስማማም 3. ምንም አልልም 4. እስማማላዉ 5. በጣም እስማማለዉ |
| 305 | በዳኒት ለመቀበል የጠበቁት ስዓት እንብዛም አልነበረም | 1. በጣም አልስማማም 2. አልስማማም 3. ምንም አልልም 4. እስማማላዉ 5. በጣም እስማማለዉ |
| 306 | የታዘዘሎትን መዳኒት እንዴት መዉሰድ እዳሎበት በተሰጦት መረጃ እረክተዋል | 1. በጣም አልስማማም 2. አልስማማም 3. ምንም አልልም 4. እስማማላዉ 5. በጣም እስማማለዉ |
| 307 | የታዘዘሎትን መዳኒት ሊያመጣ የሚችላቸዉን የጎንዮሽ ጉዳት በበቂ ዉኔታ መረጃ እግይተዋል | 1. በጣም አልስማማም 2. አልስማማም 3. ምንም አልልም 4. እስማማላዉ 5. በጣም እስማማለዉ |
| 308 | ከታዘዘሎትን መዳኒት ጋር አብረዉ መወሰድ የሌለባቸዉ መዳኒቶች፤ምግቦችና መጠጦችን በተመለከተ በበቂ ዉኔታ መረጃ እግይተዋል | 1. በጣም አልስማማም 2. አልስማማም 3. ምንም አልልም 4. እስማማላዉ 5. በጣም እስማማለዉ |

| 309 | የታዘዘሎትን መዳኒት የት መቀመጥ እንዳለበት በበቂ ዉኔታ መረጃ እግይተዋል | 1. በጣም አልስማማም 2. አልስማማም 3. ምንም አልልም 4. እስማማላዉ 5. በጣም እስማማለዉ |
| --- | --- | --- |
| 310 | በበቂ ዉኔታ ስለመዳኒቱ በጹሁፍ መረጃ አግይተዋል | 1. በጣም አልስማማም 2. አልስማማም 3. ምንም አልልም 4. እስማማላዉ 5. በጣም እስማማለዉ |
| 311 | የታዘዘልይን መዳኒት ሙሉ በሙሉ አግይቻለዉ ፤ይስማማሉ? | 1. በጣም አልስማማም 2. አልስማማም 3. ምንም አልልም 4. እስማማላዉ 5. በጣም እስማማለዉ |
| 312 | የታዘዘልይን መዳኒት ዋጋዉ ተመጣጣይ ነዉ፤ይስማማሉ? | 1. በጣም አልስማማም 2. አልስማማም 3. ምንም አልልም 4. እስማማላዉ 5. በጣም እስማማለዉ |
| 313 | የታዘዘልይን መዳኒት ጥራት ያለዉ ነዉ፤ይስማማሉ? | 1. በጣም አልስማማም 2. አልስማማም 3. ምንም አልልም 4. እስማማላዉ 5. በጣም እስማማለዉ |
| 314 | የምክር አገልግሎት የሰጦት በለሞያ በክብሮት ነዉ | 1. በጣም አልስማማም 2. አልስማማም 3. ምንም አልልም 4. እስማማላዉ 5. በጣም እስማማለዉ |
| 315 | የምክር አገልግሎት ሲያገዩ ሌላ ሰዉ በማይ ሰማበት ሁኔታ ነዉ | 1. በጣም አልስማማም 2. አልስማማም 3. ምንም አልልም 4. እስማማላዉ 5. በጣም እስማማለዉ |
| 316 | ባለሞያዉ ሞያዊ ሃለፊነቱን ተጡዋል | 1. በጣም አልስማማም 2. አልስማማም 3. ምንም አልልም 4. እስማማላዉ 5. በጣም እስማማለዉ |

| 317 | ባለሞያዉ ጥያቄዎች ለመመለስ ፍቃደያ ነዉ፤ይስማማሉ? | 1. በጣም አልስማማም 2. አልስማማም 3. ምንም አልልም 4. እስማማላዉ 5. በጣም እስማማለዉ |
| --- | --- | --- |
| 318 | ለምክር አገልግሎት ባለሞያዉ የተጠቀመዉ ግዜ በቂ ነዉ | 1. በጣም አልስማማም 2. አልስማማም 3. ምንም አልልም 4. እስማማላዉ 5. በጣም እስማማለዉ |

ክፍል-4 ስለመዳኒት ትዛዝ መገምጊያ ቅጽ

**ቦት: ______________________________________________________**

**መረጃ ስብሳቢ: _____________________________________________________ ቀን: ______**

***0=የለም and 1=አለ**

| **ተ.ቁ** | **ኮድ** | **የታዘዘበት ቀን** | **የመዳኒት ብዛት** | **በጀኔሪክ ስማ የታዘዙ የመዳኒት ቁጥር** | **አንቲባዮቲክስ**  **(0/1)*** | **በመርፌ የሚሰጡ**  **(0/1)*** | **ከመሠረታዊ ዳኒት ዝርዝር ዉስጥ የታዘዙ** | **ዋጋ** |
| --- | --- | --- | --- | --- | --- | --- | --- | --- |
|  |  |  |  |  |  |  |  |  |
|  |  |  |  |  |  |  |  |  |
|  |  |  |  |  |  |  |  |  |
|  |  |  |  |  |  |  |  |  |
|  |  |  |  |  |  |  |  |  |
|  |  |  |  |  |  |  |  |  |
|  |  |  |  |  |  |  |  |  |
|  |  |  |  |  |  |  |  |  |
|  |  |  |  |  |  |  |  |  |
|  |  |  |  |  |  |  |  |  |
|  |  |  |  |  |  |  |  |  |
|  |  |  |  |  |  |  |  |  |
|  |  |  |  |  |  |  |  |  |
|  |  |  |  |  |  |  |  |  |
|  |  |  |  |  |  |  |  |  |
|  |  |  |  |  |  |  |  |  |
|  |  |  |  |  |  |  |  |  |
|  |  |  |  |  |  |  |  |  |
|  |  |  |  |  |  |  |  |  |
|  |  |  |  |  |  |  |  |  |
|  |  |  |  |  |  |  |  |  |
|  |  |  |  |  |  |  |  |  |
|  |  |  |  |  |  |  |  |  |
|  |  |  |  |  |  |  |  |  |
|  |  |  |  |  |  |  |  |  |
|  |  |  |  |  |  |  |  |  |
|  |  |  |  |  |  |  |  |  |
|  |  |  |  |  |  |  |  |  |
|  |  |  |  |  |  |  |  |  |
|  |  |  |  |  |  |  |  |  |
|  |  |  |  |  |  |  |  |  |
| **ተቅላላ** |  |  |  |  |  |  |  |  |

**ክፍል-5 ከበሽተያ ጋር ተያያኀዝ የሆኑ መረጃዎች**

**ቦታ: ______________________________________________________**

**መለጃ ስብሳቢ: _____________________________________________________ ቀን: ______**

| **ተቁ** | **ኮድ** | **የምክር አገልግሎት በደቂቃ** | **የመዳሊት እደላ በሰከንድ** | **አጠቃላይ የታዘዘዉ መዳኒት ብዛት** | **ለበሽተያ የተሰጠዉ መዳኒት ብዛት** | **የጹሁፍ መረጃ -የመዳኒት ብዛት** |
| --- | --- | --- | --- | --- | --- | --- |
|  |  |  |  |  |  |  |
|  |  |  |  |  |  |  |
|  |  |  |  |  |  |  |
|  |  |  |  |  |  |  |
|  |  |  |  |  |  |  |
|  |  |  |  |  |  |  |
|  |  |  |  |  |  |  |
|  |  |  |  |  |  |  |
|  |  |  |  |  |  |  |
|  |  |  |  |  |  |  |
|  |  |  |  |  |  |  |
|  |  |  |  |  |  |  |
|  |  |  |  |  |  |  |
|  |  |  |  |  |  |  |
|  |  |  |  |  |  |  |
|  |  |  |  |  |  |  |
|  |  |  |  |  |  |  |
|  |  |  |  |  |  |  |
|  |  |  |  |  |  |  |
|  |  |  |  |  |  |  |
|  |  |  |  |  |  |  |
|  |  |  |  |  |  |  |
|  |  |  |  |  |  |  |
|  |  |  |  |  |  |  |
|  |  |  |  |  |  |  |
|  |  |  |  |  |  |  |
|  |  |  |  |  |  |  |
|  |  |  |  |  |  |  |
|  |  |  |  |  |  |  |
|  |  |  |  |  |  |  |
|  |  |  |  |  |  |  |
|  |  |  |  |  |  |  |
|  |  |  |  |  |  |  |
|  |  |  |  |  |  |  |
|  |  |  |  |  |  |  |

**ክፍል-6 የሆስፒታሉ መገማገሚያ ቅጽ( በእይታ የሚሞላ)**

የሆስፒታሉ ስም: ____________________________ ቀን______________

መረጃ ስብሳቢ_______________________________ መረጃ ለመሰብሰብ የፈጀዉ ግዜ: _____ ስዓት.: ደቂቃ

**ክፍል ‘ሀ’. መሠረታዊ መዳኒቶች**

| **No.** | **Medicine name** | **በመዳኒት መጋዘን ዉስጥ** | |
| --- | --- | --- | --- |
|  |  | **አለ=1** | **የለም=0** |
|  | Amoxicillin 125mg/5ml syrup/suspension |  |  |
|  | Amoxicillin 250mg/500mg cap/tab |  |  |
|  | Ceftriaxone 500mg/ 1g inj |  |  |
|  | Ciprofloxacin 500mg caps/tab |  |  |
|  | Sulphamethoxazole + Trimethoprim 200mg + 40mg in 5ml |  |  |
|  | Arthmeter + Lunfanthrine |  |  |
|  | Mebendazole oral suspension,100mg/5ml |  |  |
|  | Metronidazole 250mg cap/tab |  |  |
|  | Atenolol 50mg tab |  |  |
|  | Enalapril 5/10mg tab |  |  |
|  | Hydrochlorothiazide 25mg tab |  |  |
|  | Metformin 500mg tab |  |  |
|  | Simvastatin 20mg tab |  |  |
|  | Diazepam 5mg tab |  |  |
|  | Amitriptyline 25mg tab |  |  |
|  | Fluoxetine 20mg cap |  |  |
|  | Phenobarbitone 100mg tab |  |  |
|  | Haloperidol tab |  |  |
|  | Omeprazole 20mg cap |  |  |
|  | Salbutamol inhalers |  |  |
|  | Oral rehydration salts (ORS)/zinc |  |  |
|  | Diclofenac sodium 50mg tab |  |  |
|  | Paracetamol 120mg/5ml |  |  |
|  | Sodium chloride 0.9% (normal saline) |  |  |
|  | Oxytocin 10 IU |  |  |
|  | Magnesium sulphate inj. |  |  |
|  | Ferrous sulphate + folic acid tab |  |  |
|  | Oral contraceptives tab |  |  |
|  | Vitamin K 10 IU |  |  |
|  | Tetracycline eye ointment |  |  |

**ንዑስ ክፍል ‘ለ ‘ መሠረታዊ መዳኒቶች የሌሉት የቀናት ብዛት**

| **No.** | **Medicine name** | **የሌሉበት ቀናት** |
| --- | --- | --- |
|  | Amoxicillin 125mg/5ml syrup/suspension |  |
|  | Amoxicillin 250mg/500mg cap/tab |  |
|  | Ceftriaxone 500mg/ 1g inj |  |
|  | Ciprofloxacin 500mg caps/tab |  |
|  | Sulphamethoxazole + Trimethoprim 200mg + 40mg in 5ml |  |
|  | Arthmeter + Lunfanthrine |  |
|  | Mebendazole oral suspension,100mg/5ml |  |
|  | Metronidazole 250mg cap/tab |  |
|  | Atenolol 50mg tab |  |
|  | Enalapril 5/10mg tab |  |
|  | Hydrochlorothiazide 25mg tab |  |
|  | Metformin 500mg tab |  |
|  | Simvastatin 20mg tab |  |
|  | Diazepam 5mg tab |  |
|  | Amitriptyline 25mg tab |  |
|  | Fluoxetine 20mg cap |  |
|  | Phenobarbitone 100mg tab |  |
|  | Haloperidol tab |  |
|  | Omeprazole 20mg cap |  |
|  | Salbutamol inhalers |  |
|  | Oral rehydration salts (ORS)/zinc |  |
|  | Diclofenac sodium 50mg tab |  |
|  | Paracetamol 120mg/5ml |  |
|  | Sodium chloride 0.9% (normal saline) |  |
|  | Oxytocin 10 IU |  |
|  | Magnesium sulphate inj. |  |
|  | Ferrous sulphate + folic acid tab |  |
|  | Oral contraceptives tab |  |
|  | Vitamin K 10 IU |  |
|  | Tetracycline eye ointment |  |
